# HER2 regulates HIF-2α and drives an increased hypoxic response in breast cancer

**DOI:** 10.1186/s13058-019-1097-0

**Published:** 2019-01-22

**Authors:** Edward J. Jarman, Carol Ward, Arran K. Turnbull, Carlos Martinez-Perez, James Meehan, Chrysi Xintaropoulou, Andrew H. Sims, Simon P. Langdon

**Affiliations:** 1 Cancer Research UK Edinburgh Centre and Division of Pathology Laboratory, Institute of Genetics and Molecular Medicine, University of Edinburgh, Edinburgh, EH4 2XU UK; 2 Applied Bioinformatics of Cancer, University of Edinburgh Cancer Research Centre, MRC Institute of Genetics and Molecular Medicine, Edinburgh, EH4 2XR UK; 3 Institute of Genetics and Molecular Medicine, University of Edinburgh, Western General Hospital, Crewe Road South, Edinburgh, EH4 2XU UK

**Keywords:** Hypoxia-inducible factors, HIF-2α, HER2, Hypoxia

## Abstract

**Background:**

Tumour hypoxia is a driver of breast cancer progression associated with worse prognosis and more aggressive disease. The cellular response to hypoxia is mediated by the hypoxia-inducible transcription factors HIF-1 and HIF-2, whose transcriptional activity is canonically regulated through their oxygen-labile HIF-α subunits. These are constitutively degraded in the presence of oxygen; however, HIF-1α can be stabilised, even at high oxygen concentrations, through the activation of HER receptor signalling. Despite this, there is still limited understanding on how HER receptor signalling interacts with HIF activity to contribute to breast cancer progression in the context of tumour hypoxia.

**Methods:**

2D and 3D cell line models were used alongside microarray gene expression analysis and meta-analysis of publicly available gene expression datasets to assess the impact of HER2 overexpression on HIF-1α/HIF-2α regulation and to compare the global transcriptomic response to acute and chronic hypoxia in an isogenic cell line model of HER2 overexpression.

**Results:**

HER2 overexpression in MCF7 cells leads to an increase in HIF-2α but not HIF-1α expression in normoxia and an increased upregulation of HIF-2α in hypoxia. Global gene expression analysis showed that HER2 overexpression in these cells promotes an exaggerated transcriptional response to both short-term and long-term hypoxia, with increased expression of numerous hypoxia response genes. HIF-2α expression is frequently higher in HER2-overexpressing tumours and is associated with worse disease-specific survival in HER2-positive breast cancer patients. HER2-overexpressing cell lines demonstrate an increased sensitivity to targeted HIF-2α inhibition through either siRNA or the use of a small molecule inhibitor of HIF-2α translation.

**Conclusions:**

This study suggests an important interplay between HER2 expression and HIF-2α in breast cancer and highlights the potential for HER2 to drive the expression of numerous hypoxia response genes in normoxia and hypoxia. Overall, these findings show the importance of understanding the regulation of HIF activity in a variety of breast cancer subtypes and points to the potential of targeting HIF-2α as a therapy for HER2-positive breast cancer.

**Electronic supplementary material:**

The online version of this article (10.1186/s13058-019-1097-0) contains supplementary material, which is available to authorized users.

## Background

The rapid growth of solid tumours often results in the development of hypoxic regions. This occurs as incessant growth and vascular abnormalities lead to insufficient perfusion of the tumour mass [1, 2]. Areas of hypoxia can be found in as many as half of all breast cancers [3] and are directly linked to aggressive disease and poor response to chemo- and radiotherapy [4].

The cellular response to hypoxia is primarily mediated by the hypoxia-inducible factor (HIF) family of transcription factors. HIFs form heterodimeric transcription factors consisting of an oxygen-labile α-subunit (HIF-α) and an oxygen-independent HIF-β subunit (predominantly HIF-1β). The downstream effects of HIF activity are determined by which α subunit is involved; HIF-1α and HIF-2α have been shown to be the main drivers of the cellular response to low oxygen, whilst HIF-3α is considered a dominant negative regulator of HIF activity. Canonical regulation of hypoxia-inducible factors occurs through the oxygen-dependent proteasomal degradation of HIF-α subunits following ubiquitination by the Von Hippel-Lindau (VHL) E3 ubiquitin ligase [5]. Recognition by the VHL complex involves hydroxylation of HIF-α by the PHD family of prolyl hydroxylases, which requires molecular oxygen as a substrate [6]. In hypoxic cells, PHD-mediated hydroxylation of HIF-α is restricted by the low availability of molecular oxygen. This results in an accumulation of HIF-α subunits, which are then able to form active dimers with HIF-β and drive the transcription of hypoxia response genes [7–9].

HIF-1 activity has been associated with worse prognosis in breast cancer [10–12] and is known to drive the upregulation of genes involved in glycolysis, angiogenesis, invasion and a number of other pathologically relevant characteristics [13–16]. Whilst HIF signalling has historically been largely synonymous with HIF-1, there has been a growing appreciation of the differences between HIF-1 and HIF-2 with regard to the genes they regulate and the downstream responses mediated by their activation. More recently, analyses of HIF-1 and HIF-2 targets in breast cancer cell lines have proposed overlapping but non-redundant functions for HIF-1 and HIF-2 in breast cancer cells [17–19]. However, previous research has focussed solely on hormone-driven breast cancer cell lines and little is known about the roles of HIF-1 and HIF-2 in the context of growth factor-driven breast cancers, such as those belonging to the HER2-positive subtype.

Investigations into HIF-1α expression in breast cancers have shown correlation with the expression of HER2 [20, 21], a growth factor receptor overexpressed in 20% of breast cancers [22] and associated with more aggressive disease [23]. Additionally, growth factor signalling via the HER3 ligand neuregulin-1β has been shown to drive HIF-1α levels in normoxia through increases in protein translation and stability in breast cancer cell lines [24, 25]. This suggests that a non-canonical regulation of HIF-α subunits through growth factor signalling might promote HIF-mediated pathology in a growth factor-dependent, oxygen-independent manner. It is unclear whether growth factor-driven HIF-α regulation can broadly affect the cellular response to hypoxia, potentially acting to exacerbate tumour pathology further in hypoxic regions. Additionally, the contribution of HIF-2α to growth factor-driven HIF activity in breast cancer is not known.

In this study, we investigated the role of HER2 overexpression and growth factor-driven HIF-2α in normoxic and hypoxic breast cancer pathology. We demonstrate that HER2 overexpression enhances the cellular response to hypoxia, including the upregulation of genes implicated in driving breast cancer progression, and establish that growth factor signalling through HER2 is able to drive an oxygen-independent upregulation of HIF-2α through mechanisms different to those previously shown for HIF-1α. Finally, through HIF-2α-specific inhibition studies and survival analysis in publicly available datasets, we provide evidence that HIF-2α may represent a targetable pathway in HER2-positive breast cancer.

## Methods

### Cell lines and culture

Breast cancer cell lines were cultured in Dulbecco’s modified Eagle medium (DMEM) supplemented with 10% foetal bovine serum, 100 U/ml penicillin and 100 μg/ml streptomycin. Hypoxic cells were grown at 0.5% O_2_ in a Don Whitley H35 Hypoxystation. The genetically modified MCF7-HER2 cell line was developed in Kent Osborne’s lab and grown in media supplemented with 1 mg/ml G418 [26]. The MCF7 cell line was purchased from the ATCC, and both cell lines were tested and authenticated using STR profiling by Public Health England, Porton Down, Salisbury, UK (November 2014).

### Protein isolation

Whole cell lysates were obtained by washing cells with PBS before cellular lysis using a solution of 50 mM Tris (pH 7.5), 5 mM EGTA (pH 8.5) and 150 mM NaCl containing one complete protease inhibitor tablet (Roche), 100 μl phosphatase inhibitor cocktail 2, 100 μl phosphatase inhibitor cocktail 3, 50 μl aprotinin and 100 μl Triton X-100 per 10 ml of solution. After lysis, samples were centrifuged at 13,000*g*. Supernatants were collected and stored at − 70 °C. Each step of this procedure was performed at 4 °C.

### Gel electrophoresis and western blot analysis

Isolated proteins were separated through SDS-PAGE using a 7.5% bis-acrylamide gel. Separated proteins were transferred onto an Immobilon-P transfer PVDF membrane. The membrane was blocked and incubated overnight at 4 °C with primary antibodies (anti-HIF-1α mouse monoclonal [BD Transduction Laboratories, 610958, 1:250], anti-HIF-2α rabbit polyclonal [Novus Biologicals, NB100-122, 1:1000], anti-CAIX mouse monoclonal [Bioscience Slovakia, M75, 1:1000], anti-HER2 mouse monoclonal [Cell Signalling Technology, #2248, 1:1000], anti-pHER2 (Tyr1221/1222) rabbit monoclonal [Cell Signalling Technology, #2243, 1:1000], anti-AKT mouse monoclonal [Cell Signalling Technology, #2920, 1:1000], anti-pAKT (Ser472) rabbit polyclonal [Cell Signalling Technology. #9271, 1:1000], anti-ERK1/2 rabbit polyclonal [Cell Signalling Technology, #9102, 1:1000], anti-pERK1/2 mouse monoclonal (Thr202/Tyr204) [Cell Signalling Technology, #9106, 1:1000] and anti-α-tubulin mouse monoclonal [Abcam, Ab7291, 1:10,000]). Antibodies were detected using IRDye 800CW [Li-Cor, 926-32210, 1:10,000] and IRDye 680LT [Li-cor, 926-68021, 1:10,000] secondary antibodies with the Li-Cor Odyssey imaging system.

### Sulforhodamine B assay

Cells were seeded into 96-well plates and incubated for 24 h. Treatment was carried out for 5 days before plates were fixed by the addition of 50 μl 25% trichloroacetic acid solution per well for 1 h at 4 °C. Plates were washed with H_2_O and air dried before the addition of 50 μl 0.4% sulforhodamine B solution in 1% acetic acid for 30 min. Plates were washed in 1% acetic acid and air dried before the addition of 150 μl 10 mM Tris solution (pH 10.5). After 30 min absorbance of each well was measured at 540 nm on a BP900 biohit plate reader. HIF-2α inhibitor C76 was purchased from Merk Millipore (catalogue number 400087).

### 3D culture

Cell lines were grown as normal in 2D until 80% confluent. These were then trypsinised to form a single-cell suspension, transferred to magnetic spinner flasks (VWR) and incubated at 37 °C, 5% CO_2_. Spheroids were grown for 1–2 weeks with regular media changes until spheroids of 1–2 mm had formed. These were fixed in formalin and paraffin embedded.

### Immunohistochemistry

Paraffin-embedded spheroids were sectioned and mounted onto glass slides. Antigen retrieval was performed using a solution of 0.1 M citric acid and 0.1 M sodium citrate. Endogenous peroxidase activity was quenched using H_2_O_2_ solution. Slides were treated with total protein block (Dako) to inhibit non-specific binding and were then incubated with primary antibodies for 1 h at room temperature (anti-HIF-1α mouse monoclonal [BD Transduction Laboratories, 610958, 1:100], anti-HIF-2α rabbit polyclonal [Novus Biologicals, NB100-122, 1:200], anti-CAIX mouse monoclonal [Bioscience Slovakia, M75, 1:1000], anti-LDHA [Cell Signalling Technology, #3582, 1:400], anti-VEGF mouse monoclonal [Novus Biologicals, NB100-664, 1:50], anti-CD44 [Cell Signalling Technology, #3570, 1:50]). Slides were incubated for 30 min with Dako envision labelled polymer, before staining with Dako DAB and substrate buffer. Slides were counterstained with haemotoxylin and imaged using the Nanozoomer XR slide scanning system (Hamamatsu).

### siRNA treatment

Cells were seeded in 6-well plates (2.5 × 10^5^ per well) or in 96-well plates (1 × 10^3^ cells per well) with antibiotic-free medium for 24 h. Cells were transfected using Dharmafect transfection reagent 1 (Dharamacon) with the desired concentration of siRNA (HIF-2α siRNA [J-004814-09, 25 nM], HIF-2α pool siRNA [L-004814-00, 10 nM], non-targeting control siRNA [D-001810-10, 25 nM]). Cells were left for a further 24 h before being changed into fresh antibiotic-containing media and grown as per the experiment in normoxic (20% O_2_) or hypoxic culture conditions.

### Gene expression analysis

RNA was extracted from cells grown in normoxia or hypoxia (0.5% oxygen) for various time points using the RNeasy Mini Kit (Qiagen). Real-time PCR was performed using the StepOnePlus Real-Time PCR system (Applied Biosystems). Reverse transcription and amplification reactions were performed using the Taqman RNA-to-CT 1-Step Kit and Taqman primer sets targeting HIF-1α (Hs_00153153_m1), HIF-2α (Hs_01026149_m1) and housekeeping genes PUM1 (Hs_00472881_m1), TBP (Hs_00427620_m1) and RPL37a (Hs_01102345_m1). Gene expression values were normalised to the geometric mean of housekeeping genes.

For transcriptome analysis, RNA was extracted from cells grown in normoxia, 0.5% oxygen for 24 h (acute hypoxia), or continually cultured in 0.5% oxygen for > 10 weeks (chronic hypoxia). This was done in biological triplicate using the RNeasy Mini Kit (Qiagen). RNA was quantified using the Nanodrop 2000c system (Thermo Scientific). RNA was reverse transcribed, amplified and labelled using the Illumina TotalPrep RNA amplification kit (Ambion). Labelled RNA was hybridised to Human HT12v4 whole-genome Illumina BeadChip Arrays. Arrays were scanned using the Illumina iScan system. Raw gene expression files were filtered using the Illumina probe detection *P*-value, log_2_ transformed and quantile normalised using the lumi Bioconductor package [27]. Pairwise rank products testing (FDR < 0.05) was used to identify genes differentially expressed between cell lines or treatment groups [28]. Enrichment for gene ontology (GO) terms was tested using the online bioinformatics platform DAVID 6.7 (Database for Annotation, Visualization and Integrated Discovery) [29, 30]. Heatmaps of Log_2_ gene expression values were created in TM4 microarray software suite’s MultiExperiment Viewer [31, 32] and hierarchical clustering by Pearson correlation with complete linkage was used to compare samples. Gene expression data was uploaded to NCBI GEO under the accession GSE111246.

## Datasets

To examine gene expression across primary breast cancers, an integrated dataset of 2999 samples was interrogated. This included 17 gene expression datasets downloaded from NCBI GEO, summarised, and normalised before integration using ComBat to remove dataset-specific bias as previously described [33] (GSE12276, GSE21653, GSE3744, GSE5460, GSE2109. GSE1561, GSE17907, GSE2990, GSE7390, GSE11121, GSE16716, GSE2034, GSE1456, GSE6532, GSE3494, GSE68892). Similarly, three breast cancer cell line panel gene expression datasets containing 173 samples representing 77 different cell lines were downloaded from NCBI GEO (GSE10890, GSE12777, GSE69017) summarised, normalised and batch-corrected [33].

The METABRIC (Molecular Taxonomy of Breast cancer International Consortium) dataset is publically available and was downloaded from cbioportal.org. This dataset consists of over 2000 fresh-frozen breast cancer samples with gene expression and associated clinical data [34]. Survival analysis and Cox proportional hazards analysis were performed using R. Exhaustive Cox proportional hazards analysis across all cut points was performed using the survivALL package in R [35].

## Results

### HER2 drives HIF-2α expression in breast cancer

**Fig. 1:**
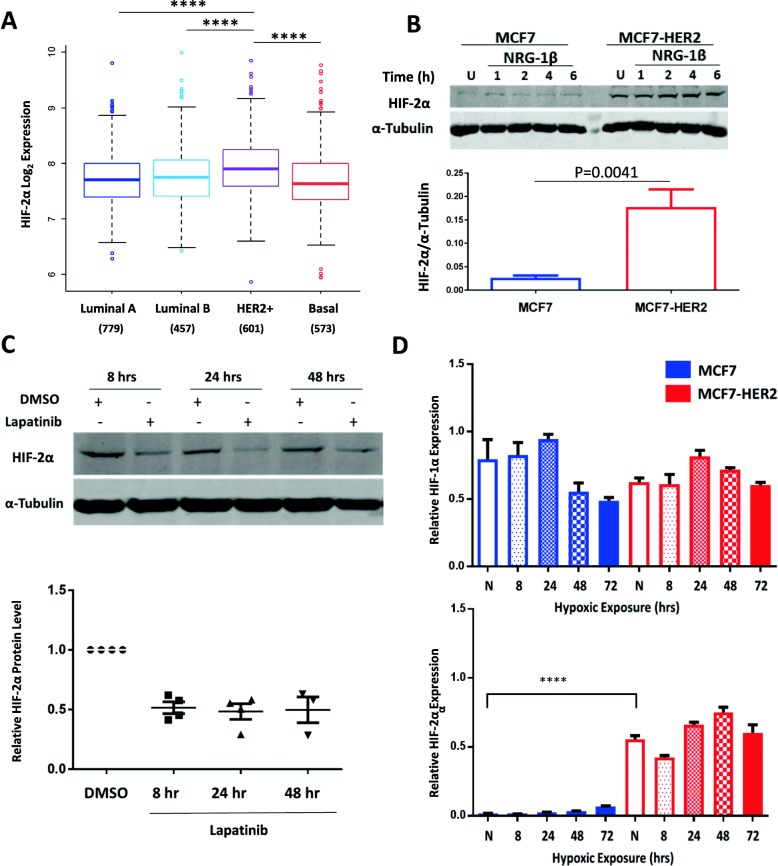
HER2 promotes HIF-2α expression in patient samples and a cell line model of HER2 overexpression. **a** HIF-2α is significantly higher in the HER2-positive subtype when compared to Luminal A, Luminal B or Basal (*P* < 0.0001, one-way ANOVA). **b** Western blotting of MCF7 and MCF7-HER2 cell lines shows increased HIF-2α levels in normoxia when HER2 is overexpressed. This was shown in untreated cells (U) and also cells treated with 200 ng/ml NRG-1β for 1–6 h. NRG-1β treatment had no discernible effect on HIF-2α levels in either cell line. The bar graph shows mean densitometry values of HIF-2α in untreated MCF7 and MCF7-HER2 whole cell lysates (*n* = 5 experiments, error bars represent SEM). HIF-2α protein level was significantly higher in MCF7-HER2 when compared to wild-type MCF7 (*P* = 0.0041, unpaired *t* test). **c** Treatment of MCF7-HER2 with 10 μM lapatinib over 8, 24 and 48 h reduces normoxic HIF-2α expression. Mean densitometry values for HIF-2α relative to DMSO controls are shown, with SEM represented by error bars and individual experimental repeats plotted. A representative western image of this result is also shown (*n* = 4). **d** RT-PCR of HIF-1α and HIF-2α expression in MCF7 and MCF7-HER2 cell lines over 72 h hypoxia (0.5% oxygen). HIF-2α but not HIF-1α expression is significantly higher in the HER2-overexpressing cell line when compared to wild-type MCF7 in normoxia (*P* < 0.0001). HIF-2α expression was also significantly higher in MCF7-HER2 than in MCF7 cells after 8, 24, 48 and 72 h in hypoxia (0.5% oxygen) (*P* < 0.0001, one-way ANOVA with Tukey’s multiple comparisons test (*n* = 3)). * = *P* < 0.05, ** = *P* < 0.01, *** = *P* < 0.005, **** = *P* < 0.0001

We performed an initial assessment of HIF-2α gene expression in a combined meta-analysis of 2999 primary breast tumours stratified by molecular subtype (details of this dataset have previously been described [33]). This demonstrated significantly higher expression of HIF-2α in the HER2-overexpressing samples when compared to Luminal A, Luminal B and Basal subtypes, (*P* < 0.0001, ANOVA with Tukey’s post-test) with no significant differences between the other subtypes (Fig. 1a), suggesting a possible role for HER2 in regulating HIF-2α expression in breast cancer. This differs to HIF-1α gene expression which is more highly expressed in more aggressive molecular subtypes, with Luminal A showing significantly lower expression than Luminal B, and both HER2-positive and Basal subtypes showing significantly higher expression than the Luminal groups (Additional file 1: Figure S1). The differences in HIF-1α and HIF-2α gene expression across molecular subtypes suggests that these factors are regulated in ways which allow subtype-specific differences in their expression, and in the case of HIF-2α this appears to be specific to the HER2-positive subtype. HIF-2α gene expression is also associated with HER2 in a dataset of 173 breast cancer cell lines samples (Additional file 2: Figure S2). HIF-2α gene expression was significantly higher in HER2-high cell lines (*n* = 52) when compared to HER2-low cell lines (*n* = 121) groups (*P* = 0.03). To investigate this further, we assessed the levels of HIF-1α and HIF-2α proteins both basally and in response to HER receptor activation using the HER3 ligand neuregulin-1β (NRG-1β). This was done in the MCF7 breast cancer cell line and MCF7-HER2, an isogenic cell line stably overexpressing the HER2 receptor [26] (Fig. 1b). Both cell lines exhibited low levels of HIF-1α in normoxic conditions (20% O_2_) but saw HIF-1α induction in normoxia after treatment with 200 ng/ml NRG-1β (Additional file 3: Figure S3A/B). This supports the earlier literature which has shown the NRG-1β-driven increase of HIF-1α in normoxia through protein stabilisation and increased translation [24, 25]. In contrast, HIF-2α protein levels did not increase after treatment with NRG-1β in either cell line, but was constitutively higher in MCF7-HER2 when compared to wild type MCF7 cells (Fig. 1b), suggesting that high HER2 expression may be associated with higher levels of HIF-2α.

To establish whether HER2 signalling was a direct driver of HIF-2α in MCF7-HER2 cells we inhibited HER signalling using lapatinib, a dual tyrosine kinase inhibitor known to inhibit the activity of both EGFR and HER2 receptors. Treatment with 10 μM lapatinib significantly reduced the normoxic expression of HIF-2α in these cells after 8, 24 and 48 h treatment (Fig. 1c), demonstrating that HER receptor signalling is required for the elevated HIF-2α protein levels seen in these cells. Next, we compared HIF-1α and HIF-2α transcript levels in MCF7 and MCF7-HER2 cells in normoxia, as well as in cells grown in hypoxia (0.5% oxygen) for 8, 24, 48 and 72 h through quantitative RT-PCR (Fig. 1d). Whilst levels of HIF-1α RNA were similar, we saw a marked increase in the expression of HIF-2α in MCF7-HER2 cells when compared to wild-type MCF7 (Fig. 1d), showing that HER2-mediated changes in HIF-2α are, at least in part, invoked at the transcriptional level. In response to hypoxia, transcriptional changes were small, HIF-1α expression peaked at 24 h in both cell lines before dropping at later time points whilst HIF-2α expression was significantly increased by 48 h hypoxia in both cell lines (*P* < 0.01). HIF-2α transcriptional changes in response to hypoxia were very small when compared to the large increase of HIF-2α seen when HER2 is overexpressed; even after 72 h in hypoxia, HIF-2α expression in MCF7 cells was at least 6-fold lower than normoxic MCF7-HER2 samples. These findings suggest that HIF-2α may be transcriptionally regulated by HER2 signalling and that this can result in an increase in HIF-2α protein even in the presence of high oxygen concentrations. Interestingly, this provides novel evidence that HIF-1α and HIF-2α may be differentially regulated by growth factor signalling and that HER2 expression may be an important regulator of HIF-2α, but not HIF-1α, in certain cellular contexts.

### HER2 overexpression in MCF7 increases the hypoxic upregulation of HIF-2α and HIF target genes

**Fig. 2:**
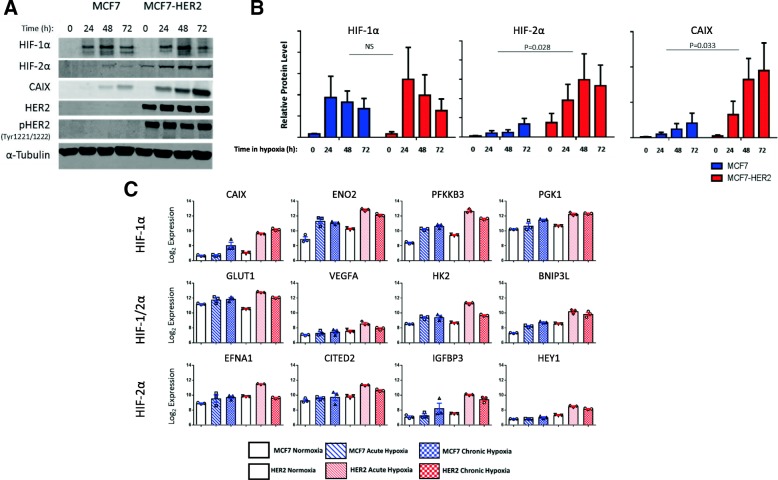
Increased upregulation of known hypoxia response genes in MCF7-HER2. Western blotting (**a**) and bar charts showing mean densitometry values (error bars = SEM) (**b**) demonstrating the increased upregulation of HIF-2α (*P* = 0.028 (*n* = 4)) and CAIX (*P* = 0.033 (*n* = 5), two-way ANOVA) in the MCF7-HER2 cell line after 24, 48 and 72 h hypoxia when compared to wild-type MCF7. No significant difference was observed for HIF-1α. **c** Gene expression data from an Illumina BeadChip Array was used to compare the expression of known HIF target genes. Four genes reported to be specific for each of HIF-1 and HIF-2 are shown alongside four genes targeted by both factors. For each gene, mean expression is shown for MCF7 and MCF7-HER2 cell lines cultured in normoxia, acute (24 h) hypoxia or chronic (> 10 weeks) hypoxia (error bars = SEM). This demonstrates the general trend toward and increased hypoxic regulation of these genes in the HER2-overexpressing cell line

Having established a role for HER2 expression and activity in driving HIF-2α transcription and protein levels in normoxia, we next assessed the effect of HER2 on the cellular response to hypoxia. For this, we used western blotting to evaluate protein levels of HIF-1α, HIF-2α and the well-characterised hypoxia response gene carbonic anhydrase IX (CAIX) after incubation in hypoxic conditions for 24, 48 and 72 h (Fig. 2a, b). In addition, we used an Illumina BeadChip HT12v4 array (NCBI GEO GSE111246) to assess global gene expression changes in these cell lines in acute (24 h 0.5% O_2_) and chronic (10 weeks 0.5% O_2_) hypoxia, comparing the expression of a number of known HIF-1 and HIF-2 target genes under these conditions (Fig. 2c).

In accordance with canonical regulatory mechanisms, hypoxia led to an increase in protein levels for both HIF-1α and HIF-2α in both cell lines. Whilst HIF-1α levels were comparable between cell lines, MCF7-HER2 had significantly higher HIF-2α levels across the hypoxic time course when compared to wild-type MCF7 cells (*P* = 0.028, two-way ANOVA). MCF7-HER2 have higher levels of HIF-2α in normoxia (in agreement with Fig. 1), and this increase in normoxic protein levels may be responsible for increased accumulation of HIF-2α in response to hypoxia. CAIX expression was also more highly upregulated in response to hypoxia in MCF7-HER2 cells when compared to the wild-type MCF7 cell line (*p* = 0.033). In addition to CAIX upregulation at the protein level, analysis of microarray gene expression data collected in normoxia (20% O_2_), acute hypoxia (24 h 0.5% O_2_) and chronic hypoxia (10 weeks 0.5% O_2_) demonstrated an increased hypoxic upregulation at the transcriptional level within the MCF7-HER2 cell line of HIF-1 genes (CAIX, ENO1, PFKFB3, PGK1) [17, 18], HIF-2-specific genes (EFNA1, CITED2, IGFBP3, HEY1) [36] and HIF-1/2-driven genes (GLUT1, VEGF,HK2, BNIP3L) [17, 18, 37, 38] previously described in the literature (Fig. 2c). In terms of HIF-2-specific genes, CITED2, IGFBP3 and HEY1 have been shown in a previous study [36] to require HIF-2α for their hypoxic upregulation in MCF7 cells, supporting the fact that these are HIF-2-mediated effects in this model. The HER2-driven expression changes of these genes in acute hypoxia are shown in Additional file 4: Figure S4. Together, we have demonstrated that HER2 overexpression in this model is able to increase the hypoxic upregulation of HIF-2α and functionally important HIF-1 and HIF-2 target genes, potentially through increased normoxic transcription of HIF-2α.

### Hypoxic response genes are more highly expressed in HER2-overexpressing 3D multicellular spheroids

**Fig. 3:**
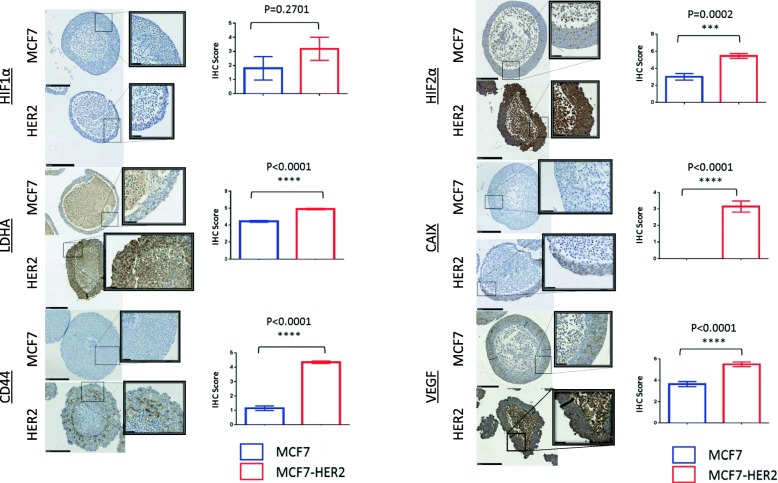
HER2 increases the levels of HIF-2α and HIF target genes in 3D spheroids. Immunohistochemistry of MCF7 and MCF7-HER2 spheroids shows the increased staining of HIF-2α, but not HIF-1α in MCF7-HER2. The increased levels of LDHA, CAIX, VEGF and CD44 were also shown. Staining was scored out of 6 for intensity and staining area. Nuclear HIF-1α spheroids were scored as a percentage of stained nuclei. Scoring for cytoplasmic or mixed staining of HIF-2α, LDHA, CAIX, CD44 and VEGF was done by first assigning a score of 0–6 for the area of staining coverage (0 = 0–10% staining, 1 = 10–25%, 2 = 25–40%, 3 = 40–55%, 4 = 55–70%, 5 = 70–85% and 6 = 85–100%) and a score of 0–3 for staining intensity, with 0 representing no visible staining, 1 being faint staining and 3 being intense staining (this was scored independently of the area covered). A final score was established by dividing the area score by 2 and adding it to the intensity score. Scoring was performed using only the viable regions of the spheroids and excluding necrotic central areas which were clearly distinguished by a loss of cellularity. Mean scoring (error bars = SEM) is shown for a minimum of seven spheroids from a representative experiment with *n* = 3 repeats. *T* tests were performed, and *P* values for the differences between cell lines are shown. (Scale bars = 250 μm (inset image scale bars = 50 μm)

Multicellular spheroids allow adherent cell lines normally grown in 2D to form 3D structures which more closely resemble in vivo tumour structures (Fig. 3). These spheroids naturally form a gradient of oxygen concentration between their necrotic central region and the well-perfused outer regions, making them a useful model for studying the effects of hypoxia in tumours and allowing these effects to be investigated with more relevance to the tumour microenvironment and tumour histology than would be possible using monolayer cell cultures.

Using multicellular spheroids of the MCF7 and MCF7-HER2 cell lines, we were able to compare the expression of hypoxia response proteins across a 3D cellular structure through immunohistochemistry. Once again, whilst the expression of HIF-1α was comparable between cell lines, HIF-2α protein levels were significantly higher in the context of HER2 overexpression (*P* = 0.0002). Increased HIF-2α expression was not limited to the hypoxic regions of the spheroids and was thus consistent with a generalised increase in the HIF-2α protein driven by increased gene expression in normoxia. Well-characterised hypoxia response genes CAIX, LDHA and VEGF showed a similar effect, with significantly increased expression across HER2-overexpressing spheroids (*P* < 0.0001 in each case). CD44, a stem cell marker and putative HIF target gene, was also significantly increased in MCF7-HER2 spheroids (*P* < 0.0001), but expression was predominantly limited to peri-necrotic, hypoxic spheroid regions, suggesting an interplay between HER2 and hypoxia in the expression of this protein. This demonstrates that HER2-mediated increases in HIF-2α and HIF target genes is also prominent in a 3D model for tumour hypoxia, but suggests that the upregulation of these hypoxic genes is promoted across a range of oxygen concentrations in this model when HER2 is overexpressed.

### Microarray gene expression analysis demonstrates a globally increased response to hypoxia in the context of HER2 overexpression

**Fig. 4:**
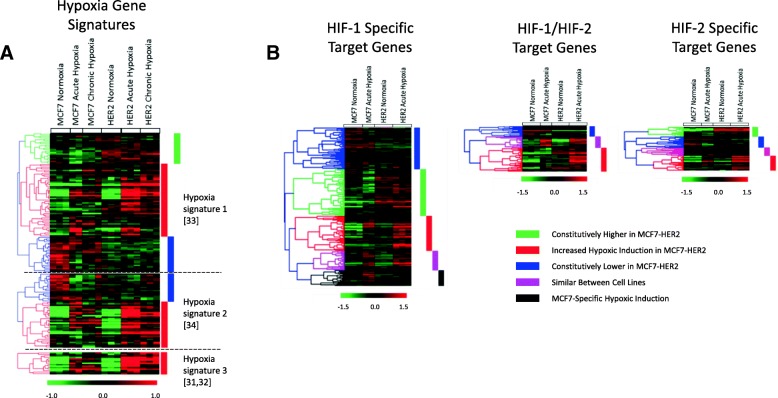
HER2 overexpression promotes the transcriptional upregulation of hypoxia metagenes and HIF target genes in hypoxia. **a** Three hypoxic gene signatures (top: Winter et al. (2007), middle: Buffa et al. (2010), bottom: Toustrup et al. (2011)) reported to show genes commonly altered by hypoxia were assessed in microarray gene expression data of MCF7 and MCF7-HER2 cultured in normoxia, acute hypoxia or chronic hypoxia. In general, MCF7-HER2 showed a stronger induction of a large proportion of genes upregulated in acute and chronic hypoxia (red clusters), with no equivalent seen for MCF7 cells. In addition, the larger signatures contained clusters which appeared to be downregulated by hypoxia in MCF7. These were constitutively lower in MCF7-HER2 (blue clusters), suggesting an increased hypoxic response in terms of both upregulated and downregulated genes. Finally, a small cluster in the largest signature (green) showed no hypoxic response, but these genes were still constitutively higher in MCF7-HER2. **b** Hierarchical clustering of HIF target genes (as determined from combined gene lists of two references Mole et al. 2009 and Schӧdel et al. 2011 who used ChIP in MCF7 to identify HIF-1 and HIF-2 target genes) was used to assess the hypoxic upregulation of HIF target genes in MCF7 and MCF7-HER2 in acute hypoxia. A large proportion of HIF targets were either constitutively higher in MCF7-HER2 (green), more strongly induced in hypoxia in MCF7-HER2 (red), or downregulated in hypoxia whilst being constitutively low in MCF7-HER2 (blue). Beside this, a small proportion behaved similarly between cell lines (pink) or were more strongly induced in MCF7 (black)

Using our Illumina BeadChip experiment (NCBI GEO GSE111246), we were able to examine transcriptomic changes in response to acute (24 h) and chronic (10 weeks) hypoxia in MCF7 and MCF7-HER2 cell lines. We used this global measurement of gene expression changes to perform a more holistic assessment of how HER2 overexpression affects cellular response to both short-term and long-term hypoxia. Initially, the transcriptional response to hypoxia was assessed through the comparison of three previously described hypoxia gene signatures [39–42] (Fig. 4a). These gene signatures have been shown to act as prognostic indicators for various cancer types including breast cancer, and together offer a robust set of genes broadly associated with hypoxia. Additionally, the upregulation of 448 HIF-1 and HIF-2 target genes previously determined through ChIP in MCF7 cells [17, 18] was assessed after acute hypoxic exposure in MCF7 and MCF7-HER2 cells (Fig. 4b). The expression of these gene sets was compared through hierarchical clustering of mean-centred log_2_ gene expression values in our cell lines (*n* = 3).

The majority of genes from the three published hypoxia gene signatures [39–42] were found to be more highly expressed in response to both acute and chronic hypoxia than normoxia (red clusters, Fig. 4a). Interestingly, the vast majority of these genes were increased to a greater extent in the MCF7-HER2 cell line when compared to wild-type MCF7. Despite no significant differences between cell lines in normoxia, expression of these genes in acute hypoxia was significantly higher in MCF7-HER2 compared to MCF7 for all three gene signatures (Winter et al., *P* = 0.0241; Buffa et al., *P* = 0.0027; Toustrup et al., *P* = 0.004), and gene expression in chronic hypoxia was significantly higher in two out of three signatures (Winter et al., *P* = 0.0032; Buffa et al., *P* = 0.0266; one-way ANOVA with Tukey’s multiple comparisons test). This suggests that HER2 can promote a stronger response to hypoxia across a broad set of hypoxia response genes when overexpressed in this cell line. Smaller gene clusters showed either reduced expression in response to hypoxia (blue clusters, Fig. 4a), or no expression change in response to hypoxia (green clusters, Fig. 4a). Interestingly, genes that were reduced in hypoxia also had constitutively lower level expression in MCF7-HER2 cells when compared to wild-type MCF7s. This suggests that HER2 overexpression is able to modulate both transcriptional up and downregulation of genes in response to hypoxia. Finally, genes showing no change in hypoxia (green cluster) were still constitutively higher in MCF7-HER2 despite no response to hypoxia, suggesting that the transcriptional regulation of hypoxic response genes which are not hypoxia-inducible in this model (due either to suboptimal conditions for activation or not being part of the cell-type-specific hypoxia transcriptional programme) may still be promoted by the increased expression of the HER2 receptor. Using the online bioinformatics tool DAVID, we assessed the enrichment of gene ontology terms relating to biological processes in these clusters and found that genes more strongly upregulated by hypoxia in MCF7-HER2 (red clusters) showed a significant increase (*P* < 0.0001) in GO (gene ontology) terms pertaining to hypoxic response and glycolysis in all three gene signatures. Genes involved included a large number of known glycolytic genes (ALDOA, PDK1, PFKFB3, PFKP, ENO1, PGK1, GPI, HK2 and PGAM1) as well as a number of non-glycolytic drivers of cancer progression (CAIX, VEGFA, LOX and ANGPTL4).

We performed hierarchical clustering analysis on a set of HIF-1 and HIF-2 target genes previously identified through ChIP in MCF7 cells (Fig. 4b) [17, 18]. These genes demonstrated similar clustering to the hypoxic signatures (Fig. 4a), with a large number of genes showing increased upregulation by acute hypoxia in the HER2-overexpressing cell line when compared to wild-type MCF7 cells (red clusters). Additionally, a large number of HIF target genes were expressed to a higher degree in normoxia in MCF7-HER2 (green clusters). The differences seen in these cell lines demonstrate an enhanced transcriptional response to hypoxia driven by HER2 overexpression. This was seen both for general hypoxia gene signatures as well as for genes shown to be HIF-1 or HIF-2 targets in MCF7 cells. These HER2-mediated transcriptional changes appear to include sets of genes whose expression in hypoxia is modulated by HER2 as well as a number of hypoxia or HIF target genes which are constitutively increased by HER2 in the presence of high oxygen concentrations. The modulation of hypoxic response genes by HER2 overexpression may therefore have important consequences for tumour progression both in the context of tumour hypoxia as well as in well-perfused tumour regions.

### HER2 overexpression in MCF7 enhances the cellular hypoxic response through primed and HER2-specific gene upregulation

**Fig. 5:**
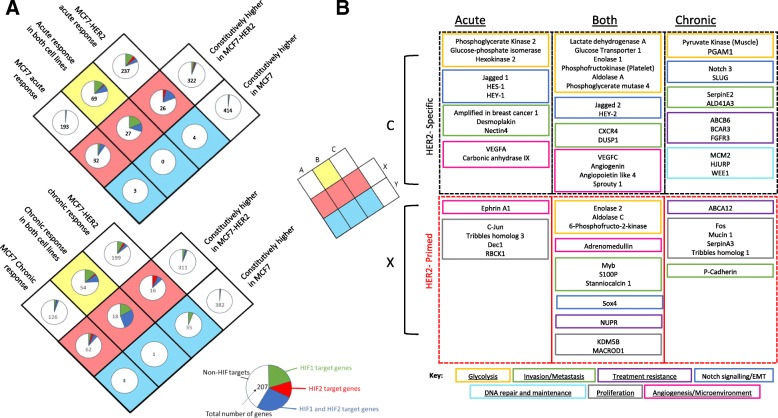
HER2 expression primes cells for hypoxia in MCF7-HER2 cells. Rank product testing of gene expression data (FDR = 0.05) was used to identify genes which were significantly induced in hypoxia (performed separately for acute and chronic) in each cell line and compared to genes significantly more highly expressed in either of the cell lines (**A**). The number of genes belonging into each category is shown in the centre of each pie chart, with the proportion of those genes represented by HIF-1, HIF-2 or HIF-1/HIF-2 target genes shown in green red or blue, respectively. Notably, a larger proportion of HIF target genes are present in hypoxic response unique to MCF7-HER2 when compared to the unique MCF7 response in acute and chronic hypoxia. Additionally, a relatively large proportion of genes which can be induced by hypoxia are constitutively increased by in MCF7-HER2, with little or no equivalent set constitutively upregulated in MCF7. This suggests that HER2 overexpression can prepare the cells for hypoxia with the normoxic expression of both HIF-regulated and non-HIF hypoxia response genes. Hypoxia response genes constitutively upregulated in MCF7-HER2 (primed genes) and genes involved in the hypoxic response in MCF7-HER2 only (HER2-specific genes) contain a number of pathologically interesting genes involved in a number of important cellular processes (**B**)

We assessed global transcriptional changes in MCF7 and MCF7-HER2 cells in response to acute and chronic hypoxia. This allowed significant gene expression changes to be identified without the use of predefined gene sets. Hypoxia-upregulated genes in each cell line were determined through rank product testing (FDR < 0.05) for both acute and chronic hypoxia. These gene lists were then compared to genes significantly upregulated in MCF7-HER2 in normoxia compared to wild-type MCF7. The genes belonging to each group are shown in a Venn diagram, with the proportion of HIF target genes in each group also shown (Fig. 5a). This analysis further demonstrates an important role for HER2 in modifying the transcriptional response to hypoxia. Notably, the number of hypoxia responsive genes which were constitutively highly expressed in MCF7-HER2 in normoxia was far greater than those constitutively higher in MCF7. Out of the genes which were constitutively highly expressed in normoxia in the context of HER2 overexpression, 14.4% were also upregulated in response to acute hypoxia and 18.6% by chronic hypoxia. This compares to just 1.2% and 7.6% for acute and chronic hypoxia respectively when genes more highly expressed in wild-type MCF7 are considered. This suggests HER2 drives the normoxic expression of genes which are upregulated in hypoxia and this is in concordance with hierarchical clustering of hypoxia gene signatures in these cell lines (Fig. 4). When genes with no significant difference in normoxic expression between cell lines are considered, we see an enrichment for HIF target genes that are upregulated by hypoxia in MCF7-HER2 but not in MCF7. HIF target genes make up just 1.6% (acute) and 5.6% (chronic) of genes induced by hypoxia specifically in MCF7 cells, compared to 19% (acute) and 14.1% (chronic) of genes induced in MCF7-HER2 cells. These findings support the gene expression changes seen through hierarchical clustering of hypoxia metagenes and HIF target genes (Fig. 4) which demonstrate a role for HER2 in driving both an exacerbated response to hypoxia as well as a normoxic increase of hypoxia-associated genes.

To more fully understand the consequences of the HER2-mediated hypoxic response, genes shown to be upregulated by hypoxia specifically in HER2-positive cells (‘HER2-specific’) and hypoxia-responsive genes shown to be more highly expressed in MCF7-HER2 than in wild-type MCF7 in normoxia (‘HER2-primed’) were assessed for known roles in breast cancer (Fig. 5b). Genes involved in glycolysis, invasion/metastasis, angiogenesis, tumour microenvironment regulation and proliferation were identified in acute hypoxia, with an additional upregulation of DNA repair/maintenance and treatment resistance genes shown in chronic hypoxia. Interestingly, a large number of genes associated with notch signalling were also found in acute and chronic hypoxia, suggesting this may promote an epithelial to mesenchymal transition phenotype in these cells under hypoxia [43–48].

**Fig. 6:**
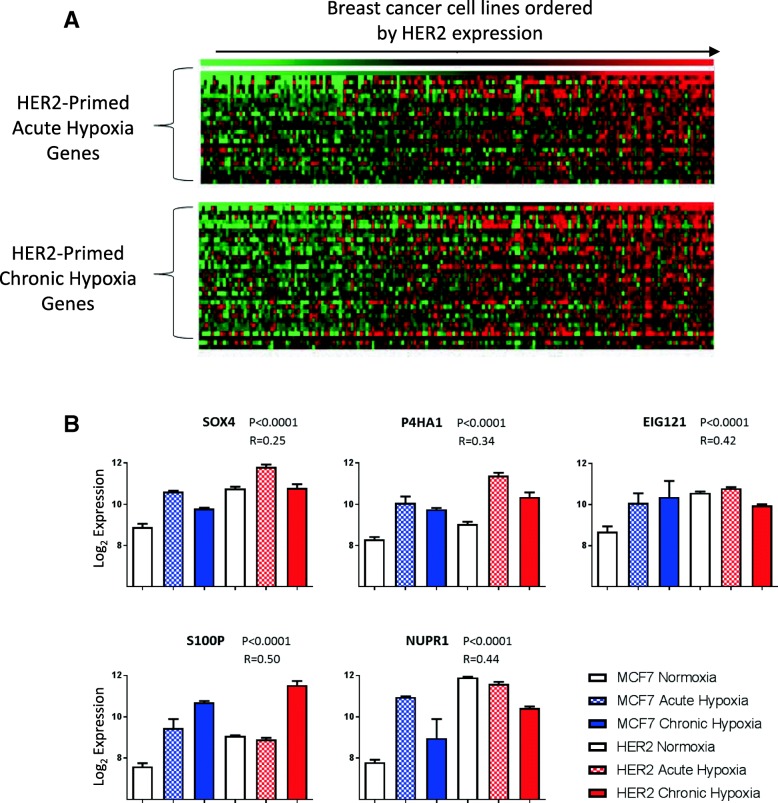
HER2 expression primes cells for hypoxia across breast cancer cell lines. **a** Genes which were primed for either acute or chronic hypoxia by HER2 overexpression in MCF7 were compared to HER2 expression in a data set containing gene expression data from 173 samples representing 77 different breast cancer cell lines. Genes with significant (*P* < 0.005) correlation to HER2 expression in this data set are shown (acute *n* = 25, chronic *n* = 33 with 16 genes represented in both acute and chronic hypoxia). The expression of these genes is shown in a heatmap with cell lines ordered by HER2 expression. **b** Five example genes with roles in cancer pathology which are primed by HER2 for both acute and chronic hypoxia and correlate to HER2 in breast cancer cell lines. Bar charts (error bars = SEM, *n* = 3) of the expression of these genes in MCF7 and MCF7-HER2 is shown to demonstrate their increased hypoxic upregulation in HER2-overexpressing cells. *P* values and Pearson’s correlation to HER2 expression in the cell lines data set are shown

To determine the relevance of this effect to other cell lines, the expression of these primed hypoxic response genes was compared to HER2 expression across a meta-analysis of gene expression data for 173 breast cancer cell line samples from 3 previously integrated studies [33] (Fig. 6a). This established a set of acute (*n* = 25) and chronic (*n* = 33) hypoxic response genes whose expression correlated significantly (*P* < 0.05) with HER2 expression across cell lines, supporting a widespread role for the HER2-driven exacerbated hypoxic response as represented by our isogenic cell line model. Figure 6b shows the top 5 acute and chronic hypoxic response genes correlating to HER2 expression: EIG121, P4HA1, S100P, NUPR1 and SOX4. Autophagy regulator EIG121 is known to promote cell survival under stress [49] and has been shown to increase metastatic capacity in breast cancer cell lines [50]. P4HA1 is a component of the prolyl-4-hydroxylase involved in collagen synthesis and ECM remodelling [51]. S100P is a calcium-binding protein with a number of cellular functions which has been directly associated with metastasis and poor survival in breast cancer [52]. NUPR1 is a stress response protein frequently amplified in breast cancer; whilst it has a large set of varied roles in the cell, its expression has been associated with progression and chemoresistance in breast cancer [53]. Finally, SOX4 is a known driver of EMT and metastatic potential in breast cancer [54, 55]. This demonstrates a strong interrelationship between HER2 expression in breast cancer cell lines and the expression of hypoxia response genes which drive metastasis and breast cancer progression, suggesting a relationship between HER2 and a response to hypoxia which is detrimental in terms of disease progression.

### HER2-overexpressing breast cancer cell lines display increased sensitivity to HIF-2 inhibition

**Fig. 7:**
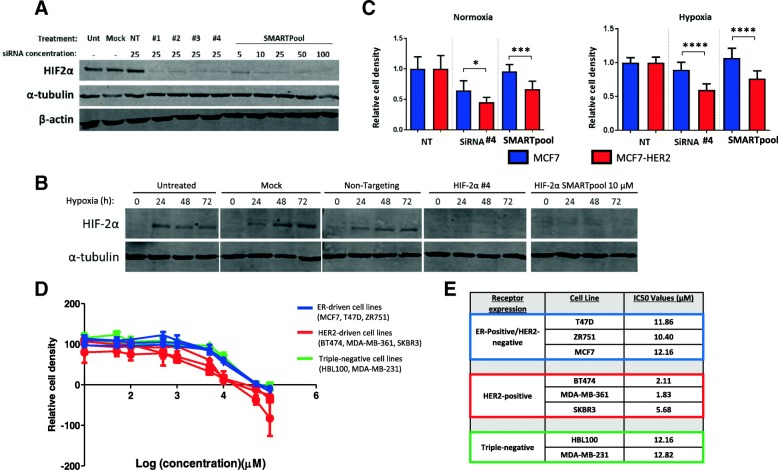
HER2-overexpressing cell lines are more sensitive to HIF-2α inhibition. **a** Western blot showing siRNAs knock-down of HIF-2α in MCF7-HER2 in normoxia. SiRNa knock-down was performed with 25 μM of four different siRNAs as well as 5–100 μM of SMARTpool, combined siRNAs. Protein level was reduced to < 10% of that in cells treated with an equivalent concentration of non-targeting siRNA up to 96 h after treatment. SiRNAs #4 and SMARTpool (10 μM) were chosen for the following experiments as HIF-2α was convincingly reduced and HIF-1α levels were not affected (data not shown). **b** Pre-treatment with either siRNA but not controls was effective at stopping the hypoxic upregulation of HIF-2α in MCF7-HER2 cells. This resulted in undetectable levels of HIF-2α protein after 24, 48 and 72 h hypoxia (0.5% oxygen). **c** MCF7 and MCF7-HER2 cells were treated with HIF-2α siRNAs and grown on 96-well plates for 5 days in either normoxia or hypoxia. Cellular density was assessed by SRB assay. Bars represent OD values relative to the non-targeting control (error bars = SEM, *n* = 12 repeats from *n* = 2 individual experiments). Differences between targeting and non-targeting siRNAs were assessed in each cell line using ANOVA with Dunnett’s multiple comparison test. In normoxia and hypoxia, MCF7-HER2 cellular growth was significantly reduced by both siRNA when compared to non-targeting siRNA (*P* < 0.0001 in all cases). MCF7 growth was reduced significantly by just one of the siRNAs in normoxia (*P* < 0.0001), and this was to a lesser extent than MCF7-HER2. In hypoxia, MCF7 was unaffected by either siRNA. MCF7-HER2 growth was inhibited to a significantly greater degree when compared to wild-type MCF7 for both siRNAs in normoxia (*P* = 0.0421 and *P* = 0.0004) and hypoxia (*P* < 0.0001 in both cases). **d** Breast cancer cell lines, including ER+/HER2- (MCF7/T47D/ZR751), HER2+ (MDAMB361, SKBR3, BT474) and triple-negative (MDAMB231 and HBL100) cells were treated for 5 days with C76 a specific inhibitor of HIF-2α translation. A range of concentrations were tested and an IC_50_ value for each cell line was established (**e**). All HER2+ cell lines had lower IC_50_ values for this compound, with no difference between ER+/HER2− and triple-negative lines

Having established a role for HER2 overexpression in driving an exacerbated hypoxic response and the increased expression of HIF-2α, we investigated whether HER2-positive cell lines were more sensitive to specific inhibition of HIF-2α. The growth of MCF7 and MCF-HER2 cell lines was compared in response to HIF-2α-specific knock-down by siRNA. Western blotting was used to confirm the HIF-2α-specific effect of two siRNA treatments; a single siRNA targeting HIF-2α (siRNA #4) and a pool of four individual HIF-2α targeting siRNAs (SMARTpool siRNA). Both treatments reduced HIF-2α to less than 10% of the level seen in untreated cells, mock transfected cells or cells treated with non-targeting siRNA; no discernible effect on HIF-1α was seen (Fig. 7a). In addition, these siRNAs were also able to reduce the levels of HIF-2α induced by hypoxia to levels below the detectable limit in MCF7-HER2 cells (Fig. 7b). Transfection of MCF7 and MCF7-HER2 cell lines with these siRNAs in sulforhodamine B (SRB) growth assays performed in normoxia or hypoxia over 5 days demonstrated an increased sensitivity in the HER2-overexpressing cell line to HIF-2α knock-down (Fig. 7c). MCF7-HER2 cells showed reduced cell density after treatment with either HIF-2α-specific siRNA in normoxia or hypoxia, whilst MCF7 cells were generally unaffected showing reduced cell density with just one of the siRNAs only in normoxia. MCF7-HER2 were significantly more sensitive to siRNA treatment than MCF7 cells in all treatment categories, indicating an increased dependence on HIF-2α in HER2-overexpressing cells in normoxia and hypoxia.

To verify the sensitivity of HER2-overexpressing breast cancer cell lines to HIF-2α-specific inhibition, we tested the effect of HIF-2α-specific translation inhibitor C76 [56] on the growth rates of eight breast cancer cell lines, including three ER-positive/HER2-negative lines (MCF7, T47D, ZR751), three naturally HER2-positive cell lines (BT474, MDA-MB-361, SKBR3) and two triple negative cell lines (HBL100, MDA-MB-231) (Fig. 7d). IC_50_ values for this compound were determined in each cell line under normoxic cell culture conditions (Fig. 7e). Non-normalised growth curves for individual cell lines are shown in Additional file 5: Figure S5. This analysis showed that all three of the HER2-positive cell lines had an increased sensitivity to HIF-2α inhibition through C76 treatment with between 1.8- and 7-fold increase in sensitivity when compared to other cell lines. IC_50_ values for HER2-positive cell lines ranged from 1.83 to 5.68 μM, with ER-positive/HER2-negative lines ranging from 10.40 to 12.16 μM and triple negative cell lines from 12.16 to 12.82 μM. This provides further evidence that HIF-2-specific inhibitors may be more effective in HER2-overexpressing breast cancers.

### High HIF-2α and HIF-2 target gene expression are indicators of poor outcome in HER2-positive breast cancer patients

**Fig. 8:**
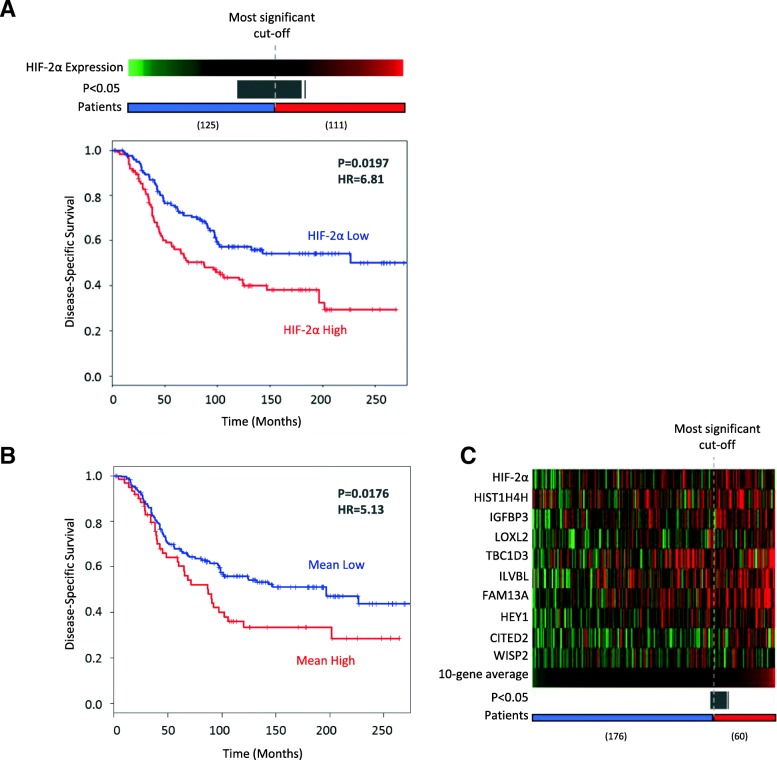
HIF-2α expression and activity is associated with worse disease-specific survival in HER2-positive breast cancer. **a** Kaplan-Meier plot showing disease-specific survival in the publically available Metabric dataset. Patients were divided into HIF-2α high and low categories using the optimum cut-off, with additional significant (*P* < 0.05) cut-offs shown represented by the grey bar. In HER2-positive breast cancers (*n* = 236) high HIF-2α expression was associated with significantly worse disease-specific survival (*P* = 0.0197, HR = 6.81). **b**, **c** A set of 9 HIF-2-specific genes reported in the literature were taken along with HIF-2α to see if their mean expression was also associated with worse survival in HER2-positive patients. Samples were ordered by their mean expression of the 10 genes and an optimum cut-off point for survival analysis was determined (**c**) (all significant cut-offs shown in grey). A high mean expression of these genes was associated with disease-specific survival in HER2-positive breast cancer (**b**)

To understand the impact of HIF-2α expression on breast cancer patient survival, we used gene expression and clinical survival data from the publicly available METABRIC [34] breast cancer dataset to assess whether HIF-2α and HIF target genes are associated with outcome. Breast cancer samples were separated into HER2-positive (*n* = 236) and HER2-negative (*n* = 1662) based on IHC-determined HER2 status. We used the survivALL R package (previously described by Pearce et al. [35]) to perform exhaustive survival analysis and obtain *P* values using Cox-proportional hazards model for every possible cut-point based on HIF-2α expression level. This demonstrated that high HIF-2α expression is associated with disease-specific survival in HER2-positive tumours (*P* = 0.0197, HR = 6.81, at the most significant cut-point). A total of 56 out of 235 cut-points (23.8%) were significant (*P* < 0.05) (grey bar in Fig. 8a), demonstrating that significant differences in survival between these groups are robust. Interestingly, HIF-2α was only associated with worse prognosis in the HER2-positive group, and not when HER2-negative, or the unstratified patient cohort is considered (Additional file 6: Figure S6). Equivalent analysis of HIF-2α and nine genes shown to be specifically driven by HIF-2 in MCF7 [36] demonstrated that when stratified by the average expression of these genes, patients with high mean expression show significantly worse disease-specific survival (*P* = 0.0176, HR = 5.13) (Fig. 8b, c). Together, this survival analysis demonstrates a robust relationship between HIF2α expression (or the expression of HIF2α-driven genes) and poor disease-specific survival in HER2-positive breast cancer patients.

## Discussion

HIF-1α expression in breast cancer is associated with more aggressive disease as HIF target genes can promote cancer cell survival in the hypoxic tumour microenvironment by regulating processes such as glycolysis, angiogenesis and metastasis [14, 57, 58]. It is now known that as well as the canonical regulatory mechanism which allows the accumulation of HIF-1α protein when molecular oxygen is limited, HIF-1α protein levels can be increased by growth factor signalling even in the presence of oxygen [24, 25]. Despite this, we still do not understand how growth factor signalling modulates the cellular response to hypoxia, or what implications this has for HER2-positive breast cancer. In this paper, we use an isogenic breast cancer cell line model for HER2 overexpression to assess the effect of increased HER2 signalling on the transcriptional response to acute (24 h) and chronic (> 10 weeks) hypoxia.

This study provides evidence for important interplay between HER2 and HIF-2α in breast cancer pathology. We have shown that HER2 overexpression is sufficient to increase cellular levels of HIF-2α but not HIF-1α in normoxia, demonstrating a novel role for the lesser studied protein. In contrast to previously described mechanisms of growth factor-regulated HIF-1α, which involves perturbations to post-translational regulation and protein stability [24, 25], this HER2-driven increase in HIF-2α was found to occur at the level of transcription and coincided with increased gene expression levels of the HIF2A gene seen in the HER2-positive subtype in a large meta-analysis of breast tumours. We have also shown that the increased expression of HER2 in these cells promotes the upregulation of HIF-2α protein in response to hypoxia, with no discernible effect on HIF-1α. HIF-2α upregulation was inhibited by treatment with lapatinib (Fig. 1c) and PI3K inhibitor LY294002 (Additional file 3: Figure S3C). Whilst the exact mechanisms governing the differential normoxic upregulation of HIF-1α and HIF-2α are still unclear, this suggests that signalling through AKT may play a role in both. HER2 overexpression also led to the increased expression of previously reported HIF-1 and HIF-2-specific target genes, and an increase in protein levels of hypoxia response genes in a 3D model which recapitulates the oxygen gradient seen in the tumour microenvironment. This included proteins previously described as HIF-1-specific targets, such as carbonic anhydrase IX (CAIX) [59] and lactate dehydrogenase A (LDHA) [14, 60]. Together, this not only provides novel evidence that the non-canonical regulation of HIF-α subunits extends to HIF-2α, but suggests that an increase in HIF-2α levels through such mechanisms is sufficient in driving the hypoxic expression of various hypoxic response genes.

We propose that the potential for the different HIF-α family members to drive the transcription of unique target genes may be plastic and context-specific and that the regulation of HIF-1α and HIF-2α levels by growth factor signalling may be an important mechanism by which the expression of numerous hypoxia response genes is modified. Previous efforts to define the differences between the transcriptional targets of HIF-1 and HIF-2 in breast cancer have focussed on using the MCF7 cell line as a model [17, 18, 36], but further work may be required to define HIF targets and the differences in HIF target selection in various breast cancer types in order to understand when targeting HIF activity may be an effective therapeutic strategy.

We have shown a generalised increase in the expression of hypoxia response genes under acute and chronic hypoxia when HER2 is overexpressed. This included the increased upregulation of previously characterised hypoxic response gene signatures, which have been shown to have prognostic potential in multiple cancer types including breast cancer [39–42]. The HER2-mediated increase in the expression of these genes in hypoxia suggests that the increased aggressiveness of HER2-positive breast cancer may in part be due to the increased potential for these cells to respond to hypoxic stress. Furthermore, by comparing global gene expression changes in response to hypoxia, we have shown that HER2 overexpression in MCF7 promotes the constitutive normoxic expression of genes also induced by hypoxia. In this way, we demonstrate the ability of HER2 expression to not only exacerbate the cellular response to hypoxia, but to promote a primed state, whereby numerous pathologically relevant hypoxia response genes (including identified HIF-1 and HIF-2 target genes) are driven in normoxia. We also found a number of these ‘primed’ hypoxic response genes to be correlated with HER2 expression across breast cancer cell lines, suggesting that the HER2-mediated upregulation of these genes is not unique to our isogenic cell line model. This analysis included a number of genes known to be involved in cell motility, invasion and metastasis, suggesting that the increase in HIF2A expression by HER2 may drive these characteristics in patients with HER2-positive breast cancers and may explain the relationship between HIF2A expression and survival in these patients.

Finally, we investigated the potential for HIF-2α-targeted therapy in HER2-positive breast cancer. Whilst HIF-1α expression has been consistently associated with disease progression and worse prognosis in breast cancer [10, 20, 61–63], the prognostic implications of HIF-2α expression have remained unclear [64, 65]. Here, we show that when survival analysis of the METABRIC patient cohort is performed in HER2-positive breast cancer patients only, HIF-2α expression and activity is clearly associated with worse disease-specific survival. We also demonstrated an increased sensitivity of HER2-positive breast cancer cell lines to HIF-2α-specific inhibition, using siRNA and the previously described HIF-2α translation inhibitor C76 [56]. This suggests that in HER2-positive breast cancers targeting HIF-2α activity may be an effective therapeutic intervention and warrants further investigation. The efficacy of HIF-targeted therapy is currently being investigated in a number of cancer types, and thus a number of compounds which target HIF activity either directly or indirectly have been developed [66, 67]. This has included an increased recognition of the important differences between HIF-1 and HIF-2 inhibition and the development of HIF-2-specific inhibitors [56, 68]. However, the application of these compounds in breast cancer therapy to date is highly limited and the majority of these therapeutic approaches have focussed entirely on HIF-1 inhibition, with the efficacy of HIF-2 inhibition being largely limited to renal cell carcinoma [69–72].

## Conclusions

In this study, we have shown that HER2 overexpression in MCF7 cells leads to an increase in HIF-2α levels in normoxia and the increased upregulation of HIF-2α in response to hypoxia; this demonstrates novel mechanistic differences in the growth factor-mediated regulation of HIF-1α and HIF-2α in breast cancer cells. In addition, we have shown that HER2 overexpression in this model drives the expression of a large number of hypoxic response genes both in normoxia and in response to acute (24 h) and chronic (10 weeks) hypoxia, suggesting that growth factor signalling may exacerbate the cellular response to hypoxia through HIF-2α. We have not only demonstrated a direct relationship between HER2 overexpression and HIF-2α in breast cancer cell lines and patient gene expression data, but have shown both an increased sensitivity of HER2-positive cell lines to HIF-2-specific inhibition and demonstrated the ability of HIF-2α- or HIF-2-specific target genes to act as prognostic indicators for disease-specific survival in HER2-positive breast cancers. This shows that HER2 expression may be an important determinant in the role of HIF-2 in breast cancer pathology and that the effectiveness of HIF-targeted therapies for breast cancer may depend on the expression of growth factor receptors such as HER2. Further investigation into the mechanisms governing the HER2-mediated regulation of HIF transcription factors is needed to fully understand the contribution of HIF-driven transcription to cancer progression in normoxia and hypoxia and may lead to new insights into how specific and non-specific HIF-targeted therapeutics can be more effectively applied clinically to the treatment of breast cancer.

## Additional file

Additional file 1: **Figure S1.** HIF-1α and HIF-2α expression across the molecular subtypes of breast cancer. HIF1A (A) and HIF2A (B) gene expression in a combined meta-analysis of 2999 breast cancer patient patients stratified by molecular subtype. Boxes represent the median, upper and lower quartiles, whilst range is represented by the whiskers. The number of patients attributed to each category is shown below the molecular subtype. ANOVA with Tukey’s multiple comparisons shows significantly higher expression of HIF1A in more aggressive subtypes whilst HIF2A is more highly expressed in the HER2-positive subtype only. Adjusted *P* values are shown on the right hand side. (PDF 153 kb)

Additional file 2: **Figure S2.** HIF2A expression is higher in cell lines with high HER2 expression. A cell line data set containing 173 samples representing 77 different breast cancer cell lines was used to compare HIF2A with HER2 expression. A) Box plot showing the expression of HIF2A in HER2-low (*n* = 121) and HER2-high (*n* = 52) cell line samples. HER2-high cell lines have significantly higher levels of HIF2A expression (*P* = 0.03, Wilcoxon signed-rank test). B) Cell lines ordered by HER2 expression to show the cut-off used to determine HER2-high and HER2-low groups. (PDF 181 kb)

Additional file 3: **Figure S3.** Regulation of HIF-2α by AKT and ERK signalling pathways. A) MCF7 and MCF7-HER2 cells show similar increases in nuclear HIF-1α after treatment with 200 ng/ml NRG-1β. HIF-1α protein levels were compared by western blotting of nuclear and cytoplasmic lysates collected from cells treated with NRG-1β for 1–6 h after 20 h in 0% FCS phenol red-free media. Β-actin is included as a loading control. B) Western blotting experiments show the increase in AKT(S473) and ERK1/2(T202/Y204) phosphorylation in response to treatment with 200 ng/ml NRG-1β in MCF7 and MCF7-HER2 cells. Levels of phosphorylated AKT and ERK1/2 are comparable between cell lines both in untreated cells and after NRG-1β treatment (representative from *n* = 4 experimental repeats. C) MCF7-HER2 cells were treated with PI3K inhibitor LY294002 (10 μM) or dual specificity MEK kinase inhibitor PD98059 (50 μM) for 8 h before whole lysate collection. Blotting for phosphorylated ERK1/2 (T202/Y204) and AKT (S473) demonstrate the specific inhibition of these pathways by their respective inhibitors. D) OD measurements of HIF-2α protein levels in LY294002 and PD98059 treated lysates compared to equivalent vehicle (DMSO) controls in western blotting experiments shown in C. HIF-2α levels were significantly reduced after PI3K inhibition (ratio pair t-test, *P* < 0.05).Whilst inhibition of ERK phosphorylation led to a reduction in HIF-2α levels in all experimental repeats, this did not achieve significance (representative of n = 4 experimental repeats). (PDF 157 kb)

Additional file 4: **Figure S4.** HER2-driven hypoxic expression of HIF-2α-specific target genes. The expression levels of IGFBP3, CITED2 and HEY1 in MCF7 and MCF7-HER2 in normoxia and after exposure to acute (24 h) hypoxia in our Illumina Beadchip microarray data. Individual expression values for *n* = 3 repeats is shown with mean values represented by blue (MCF7) or red (MCF7-HER2) lines. These target genes have been previously demonstrated by siRNA inhibition of HIF-1α and HIF-2α to be specifically upregulated by HIF-2α in MCF7 cells [28]. The upregulation of gene expression in response to acute hypoxia is greatly facilitated by the overexpression of HER2. (ANOVA with Tukey’s multiple comparisons, *** = *P* < 0.001). (PDF 13 kb)

Additional file 5: **Figure S5.** Cell growth assays show increased sensitivity of HER2-positive cell lines to HIF-2α inhibition. Sulforhodamine B growth assays for cell lines treated with a range of concentrations of HIF-2 translation inhibitor C76 or vehicle control (DMSO). The data shown is the non-normalised version of the growth curves shown in Fig. 7D. Briefly, cells were treated with 10 nM–100 μM of C76 or equivalent volumes of DMSO for 5 days, at which point cellular density was assessed by SRB assay. Error bars represent the SEM from *n* = 6 repeats. (PDF 51 kb)

Additional file 6: **Figure S6.** HIF2a is associated with worse prognosis only in HER2-positive breast cancer. Kaplain-Meier plots showing disease-specific survival in the METABRIC dataset in all patients (A), HER2-positive patients (B) and HER2-negative patients (C). HIF-2α high and low categories were determined by the optimum cut-off point, whilst the complete data set used median HIF-2α expression, as no suitable significant cut-offs were present. All significant cut-off points are illustrated by grey bars above the plots, with the number of patients in each category shown as blue and red bars. HER2 positivity was determined by IHC or FISH in associated clinical metadata. HIF-2α was only significantly associated with worse prognosis in HER2-positive patients. (PDF 290 kb)

## Abbreviations

CAIX: Carbonic anhydrase 9
EMT: Epithelial-mesenchymal transition
ER: Oestrogen receptor
HER2: Human epidermal growth factor receptor 2
HIF-1α: Hypoxia-inducible factor 1 alpha
HIF-2α: Hypoxia-inducible factor 2 alpha
LDHA: Lactate dehydrogenase A
siRNA: Small interfering RNA
SRB: Sulforhodamine B
VHL: Von Hippel-Lindau tumour suppressor

## References

[1] Brown JM, Giaccia AJ (1998). The unique physiology of solid tumors: opportunities (and problems) for cancer therapy. Cancer Res.

[2] Wilson WR, Hay MP (2011). Targeting hypoxia in cancer therapy. Nat Rev Cancer.

[3] Vaupel P, Briest S, Hockel M (2002). Hypoxia in breast cancer: pathogenesis, characterization and biological/therapeutic implications. Wien Med Wochenschr.

[4] Vaupel P, Thews O, Hoeckel M (2001). Treatment resistance of solid tumors*: role of hypoxia and anemia*. Med Oncol.

[5] Ivan M (2001). HIFalpha targeted for VHL-mediated destruction by proline hydroxylation: implications for O2 sensing. Science.

[6] Lando D (2003). Oxygen-dependent regulation of hypoxia-inducible factors by prolyl and asparaginyl hydroxylation. Eur J Biochem.

[7] Semenza GL (2001). HIF-1 and mechanisms of hypoxia sensing. Curr Opin Cell Biol.

[8] Semenza GL (2004). Hydroxylation of HIF-1: oxygen sensing at the molecular level. Physiology (Bethesda).

[9] Ward C (2013). New strategies for targeting the hypoxic tumour microenvironment in breast cancer. Cancer Treat Rev.

[10] Wang W (2014). Hypoxia-inducible factor 1alpha in breast cancer prognosis. Clin Chim Acta.

[11] Vleugel MM (2005). Differential prognostic impact of hypoxia induced and diffuse HIF-1alpha expression in invasive breast cancer. J Clin Pathol.

[12] Gruber G (2004). Hypoxia-inducible factor 1 alpha in high-risk breast cancer: an independent prognostic parameter?. Breast Cancer Res.

[13] Pugh CW, Ratcliffe PJ (2003). Regulation of angiogenesis by hypoxia: role of the HIF system. Nat Med.

[14] Semenza GL (2010). HIF-1: upstream and downstream of cancer metabolism. Curr Opin Genet Dev.

[15] Ward C (2015). Evaluation of carbonic anhydrase IX as a therapeutic target for inhibition of breast cancer invasion and metastasis using a series of in vitro breast cancer models. Oncotarget.

[16] Munoz-Najar UM (2006). Hypoxia stimulates breast carcinoma cell invasion through MT1-MMP and MMP-2 activation. Oncogene.

[17] Mole DR (2009). Genome-wide association of hypoxia-inducible factor (HIF)-1alpha and HIF-2alpha DNA binding with expression profiling of hypoxia-inducible transcripts. J Biol Chem.

[18] Schodel J (2011). High-resolution genome-wide mapping of HIF-binding sites by ChIP-seq. Blood.

[19] Keith B, Johnson RS, Simon MC (2011). HIF1alpha and HIF2alpha: sibling rivalry in hypoxic tumour growth and progression. Nat Rev Cancer.

[20] Bos R (2003). Levels of hypoxia-inducible factor-1alpha independently predict prognosis in patients with lymph node negative breast carcinoma. Cancer.

[21] Kronblad A (2006). Hypoxia inducible factor-1alpha is a prognostic marker in premenopausal patients with intermediate to highly differentiated breast cancer but not a predictive marker for tamoxifen response. Int J Cancer.

[22] Gutierrez C, Schiff R (2011). HER2: biology, detection, and clinical implications. Arch Pathol Lab Med.

[23] Menard S (2001). HER2 as a prognostic factor in breast cancer. Oncology.

[24] Laughner E (2001). HER2 (neu) signaling increases the rate of hypoxia-inducible factor 1alpha (HIF-1alpha) synthesis: novel mechanism for HIF-1-mediated vascular endothelial growth factor expression. Mol Cell Biol.

[25] Li YM (2005). A hypoxia-independent hypoxia-inducible factor-1 activation pathway induced by phosphatidylinositol-3 kinase/Akt in HER2 overexpressing cells. Cancer Res.

[26] Benz CC (1992). Estrogen-dependent, tamoxifen-resistant tumorigenic growth of MCF-7 cells transfected with HER2/neu. Breast Cancer Res Treat.

[27] Du P, Kibbe WA, Lin SM (2008). lumi: a pipeline for processing Illumina microarray. Bioinformatics.

[28] Breitling R (2004). Rank products: a simple, yet powerful, new method to detect differentially regulated genes in replicated microarray experiments. FEBS Lett.

[29] Huang da W, Sherman BT, Lempicki RA (2009). Systematic and integrative analysis of large gene lists using DAVID bioinformatics resources. Nat Protoc.

[30] Huang da W, Sherman BT, Lempicki RA (2009). Bioinformatics enrichment tools: paths toward the comprehensive functional analysis of large gene lists. Nucleic Acids Res.

[31] Saeed AI (2006). TM4 microarray software suite. Methods Enzymol.

[32] Saeed AI (2003). TM4: a free, open-source system for microarray data management and analysis. Biotechniques.

[33] Moleirinho S (2013). KIBRA exhibits MST-independent functional regulation of the Hippo signaling pathway in mammals. Oncogene.

[34] Curtis C (2012). The genomic and transcriptomic architecture of 2,000 breast tumours reveals novel subgroups. Nature.

[35] Pearce DA, et al. Continuous biomarker assessment by exhaustive survival analysis. bioRxiv. 2017. 10.1101/208660.

[36] Aprelikova O (2006). Role of ETS transcription factors in the hypoxia-inducible factor-2 target gene selection. Cancer Res.

[37] Forsythe JA (1996). Activation of vascular endothelial growth factor gene transcription by hypoxia-inducible factor 1. Mol Cell Biol.

[38] Ratcliffe PJ (2007). HIF-1 and HIF-2: working alone or together in hypoxia?. J Clin Invest.

[39] Toustrup K (2011). Development of a hypoxia gene expression classifier with predictive impact for hypoxic modification of radiotherapy in head and neck cancer. Cancer Res.

[40] Toustrup K (2012). Hypoxia gene expression signatures as prognostic and predictive markers in head and neck radiotherapy. Semin Radiat Oncol.

[41] Winter SC (2007). Relation of a hypoxia metagene derived from head and neck cancer to prognosis of multiple cancers. Cancer Res.

[42] Buffa FM (2010). Large meta-analysis of multiple cancers reveals a common, compact and highly prognostic hypoxia metagene. Br J Cancer.

[43] Sahlgren C (2008). Notch signaling mediates hypoxia-induced tumor cell migration and invasion. Proc Natl Acad Sci U S A.

[44] Chen J (2010). Hypoxia potentiates Notch signaling in breast cancer leading to decreased E-cadherin expression and increased cell migration and invasion. Br J Cancer.

[45] Wang Z (2010). The role of Notch signaling pathway in epithelial-mesenchymal transition (EMT) during development and tumor aggressiveness. Curr Drug Targets.

[46] Shao S (2015). Notch1 signaling regulates the epithelial-mesenchymal transition and invasion of breast cancer in a Slug-dependent manner. Mol Cancer.

[47] Kim RK (2016). Radiation driven epithelial-mesenchymal transition is mediated by Notch signaling in breast cancer. Oncotarget.

[48] Zhang L (2017). Activation of Notch pathway is linked with epithelial-mesenchymal transition in prostate cancer cells. Cell Cycle.

[49] Deng L, Feng J, Broaddus RR (2010). The novel estrogen-induced gene EIG121 regulates autophagy and promotes cell survival under stress. Cell Death Dis.

[50] Bauer M, Aust G, Schumacher U (2004). Different transcriptional expression of KIAA1324 and its splicing variants in human carcinoma cell lines with different metastatic capacity. Oncol Rep.

[51] Gilkes DM (2013). Hypoxia-inducible factor 1 (HIF-1) promotes extracellular matrix remodeling under hypoxic conditions by inducing P4HA1, P4HA2, and PLOD2 expression in fibroblasts. J Biol Chem.

[52] Wang G (2006). Induction of metastasis by S100P in a rat mammary model and its association with poor survival of breast cancer patients. Cancer Res.

[53] Chowdhury UR (2009). Emerging role of nuclear protein 1 (NUPR1) in cancer biology. Cancer Metastasis Rev.

[54] Zhang J (2012). SOX4 induces epithelial-mesenchymal transition and contributes to breast cancer progression. Cancer Res.

[55] Parvani JG, Schiemann WP (2013). Sox4, EMT programs, and the metastatic progression of breast cancers: mastering the masters of EMT. Breast Cancer Res.

[56] Zimmer M (2008). Small-molecule inhibitors of HIF-2a translation link its 5'UTR iron-responsive element to oxygen sensing. Mol Cell.

[57] Semenza GL (2016). The hypoxic tumor microenvironment: a driving force for breast cancer progression. Biochim Biophys Acta.

[58] Semenza GL (2012). Hypoxia-inducible factors: mediators of cancer progression and targets for cancer therapy. Trends Pharmacol Sci.

[59] Kaya AO (2012). Hypoxia inducible factor-1 alpha and carbonic anhydrase IX overexpression are associated with poor survival in breast cancer patients. J BUON.

[60] Sorensen BS (2005). Influence of oxygen concentration and pH on expression of hypoxia induced genes. Radiother Oncol.

[61] Dales JP (2005). Overexpression of hypoxia-inducible factor HIF-1alpha predicts early relapse in breast cancer: retrospective study in a series of 745 patients. Int J Cancer.

[62] Schindl M (2002). Overexpression of hypoxia-inducible factor 1alpha is associated with an unfavorable prognosis in lymph node-positive breast cancer. Clin Cancer Res.

[63] Generali D (2006). Hypoxia-inducible factor-1alpha expression predicts a poor response to primary chemoendocrine therapy and disease-free survival in primary human breast cancer. Clin Cancer Res.

[64] Helczynska K (2008). Hypoxia-inducible factor-2alpha correlates to distant recurrence and poor outcome in invasive breast cancer. Cancer Res.

[65] Stiehl DP (2012). Non-canonical HIF-2alpha function drives autonomous breast cancer cell growth via an AREG-EGFR/ErbB4 autocrine loop. Oncogene.

[66] Hu Y, Liu J, Huang H (2013). Recent agents targeting HIF-1alpha for cancer therapy. J Cell Biochem.

[67] Paolicchi E (2016). Targeting hypoxic response for cancer therapy. Oncotarget.

[68] Scheuermann TH (2013). Allosteric inhibition of hypoxia inducible factor-2 with small molecules. Nat Chem Biol.

[69] Chen W (2016). Targeting renal cell carcinoma with a HIF-2 antagonist. Nature.

[70] Burkitt K (2009). Targeting both HIF-1 and HIF-2 in human colon cancer cells improves tumor response to sunitinib treatment. Mol Cancer Ther.

[71] Yu T, Tang B, Sun X (2017). Development of inhibitors targeting hypoxia-inducible factor 1 and 2 for cancer therapy. Yonsei Med J.

[72] Burroughs SK (2013). Hypoxia inducible factor pathway inhibitors as anticancer therapeutics. Future Med Chem.

