# Supplementary material for: HER2 regulates HIF-2α and drives an increased hypoxic response in breast cancer
Source: Breast Cancer Res. 2019 Jan 22;21:10. doi: 10.1186/s13058-019-1097-0 (PMC6343358; doi:10.1186/s13058-019-1097-0)
Supplement: Supplementary file 1 — Figure S1. HIF-1α and HIF-2α expression across the molecular subtypes of breast cancer. HIF1A (A) and HIF2A (B) gene expression in a combined meta-analysis of 2999 breast cancer patient patients stratified by molecular subtype. Boxes represent the median, upper and lower quartiles, whilst range is represented by the whiskers. The number of patients attributed to each category is shown below the molecular subtype. ANOVA with Tukey’s multiple comparisons shows significantly higher expression of HIF1A in more aggressive subtypes whilst HIF2A is more highly expressed in the HER2-positive subtype only. Adjusted P values are shown on the right hand side. (PDF 153 kb) [file 13058_2019_1097_MOESM1_ESM.pdf]

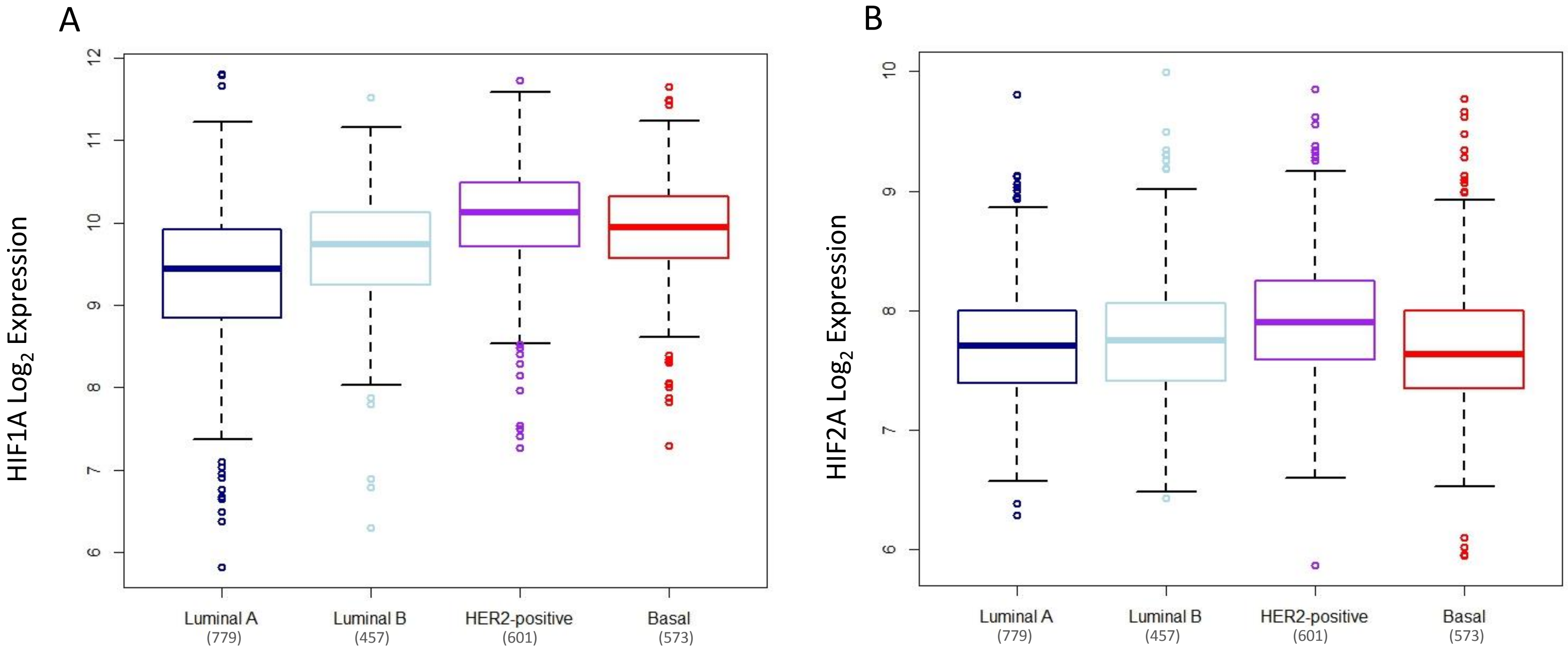

HIF1a

	p adj
Luminal B-Luminal A	0.0000000
HER2-positive-Luminal A	0.0000000
Basal-Luminal A	0.0000000
HER2-positive-Luminal B	0.0000000
Basal-Luminal B	0.0000000
Basal-HER2-positive	0.0043644

HIF2a

	p adj
Luminal B-Luminal	0.3168259
HER2-positive-Luminal A	0.0000000
Basal-Luminal A	0.9273067
HER2-positive-Luminal B	0.0000002
Basal-Luminal B	0.1379610
Basal-HER2-positive	0.0000000
