# Supplementary figures and images for: HER2 regulates HIF-2α and drives an increased hypoxic response in breast cancer
Source: Breast Cancer Res. 2019 Jan 22;21:10. doi: 10.1186/s13058-019-1097-0 (PMC6343358; doi:10.1186/s13058-019-1097-0)

**A**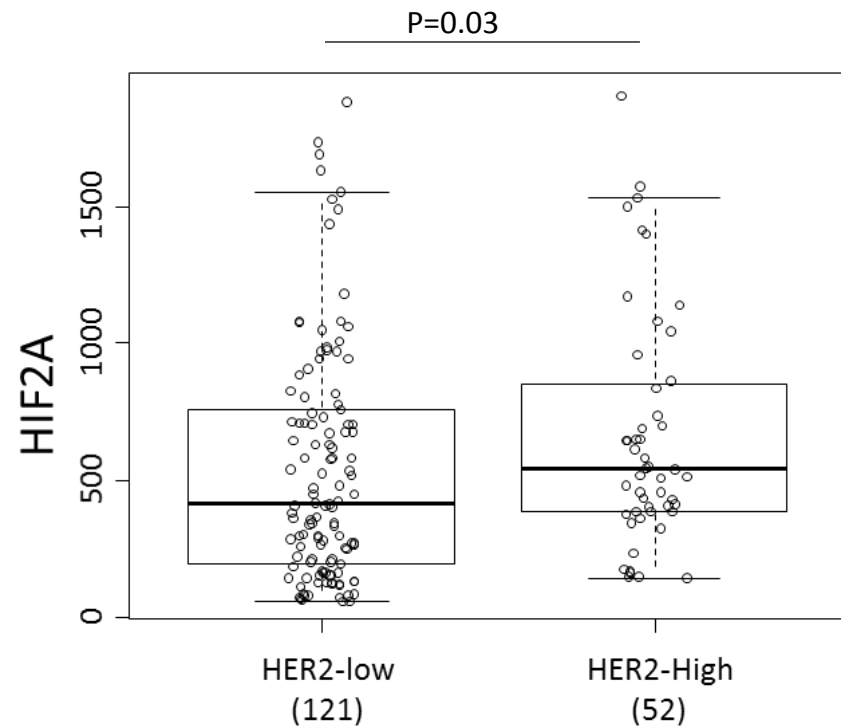**B**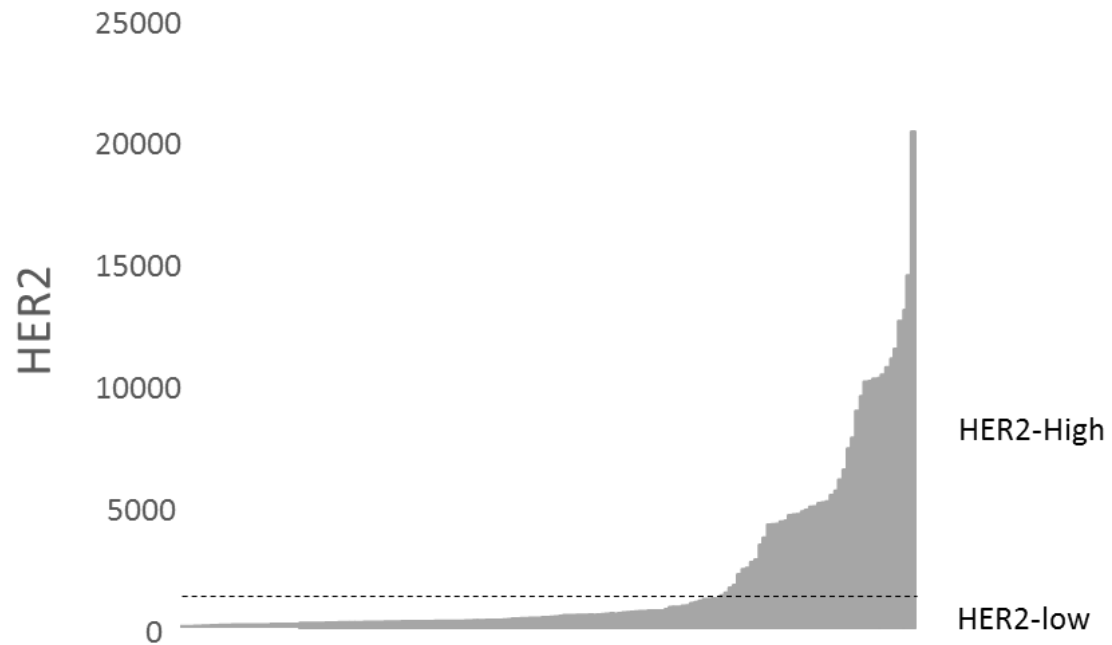

Supplement: Supplementary file 2 — Figure S2. HIF2A expression is higher in cell lines with high HER2 expression. A cell line data set containing 173 samples representing 77 different breast cancer cell lines was used to compare HIF2A with HER2 expression. A) Box plot showing the expression of HIF2A in HER2-low (n = 121) and HER2-high (n = 52) cell line samples. HER2-high cell lines have significantly higher levels of HIF2A expression (P = 0.03, Wilcoxon signed-rank test). B) Cell lines ordered by HER2 expression to show the cut-off used to determine HER2-high and HER2-low groups. (PDF 181 kb) [file 13058_2019_1097_MOESM2_ESM.pdf]

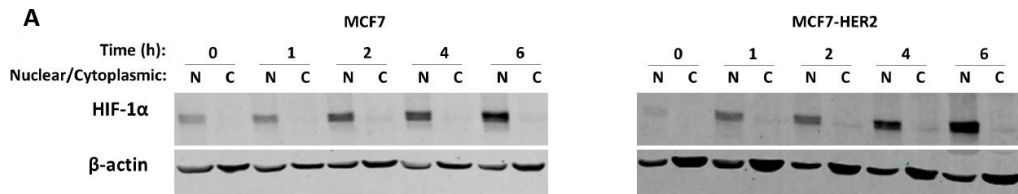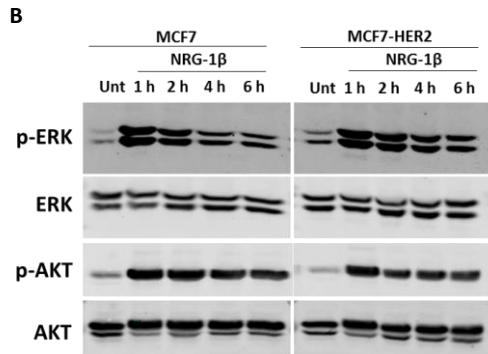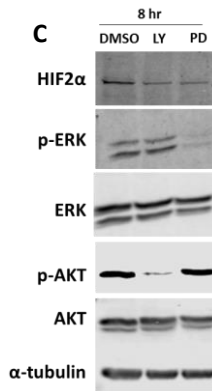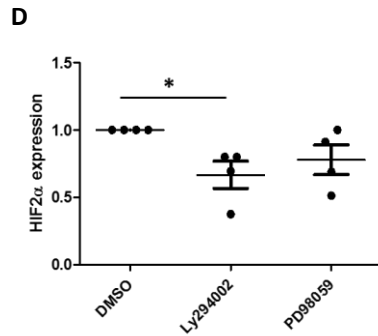

Supplement: Supplementary file 3 — Figure S3. Regulation of HIF-2α by AKT and ERK signalling pathways. A) MCF7 and MCF7-HER2 cells show similar increases in nuclear HIF-1α after treatment with 200 ng/ml NRG-1β. HIF-1α protein levels were compared by western blotting of nuclear and cytoplasmic lysates collected from cells treated with NRG-1β for 1–6 h after 20 h in 0% FCS phenol red-free media. Β-actin is included as a loading control. B) Western blotting experiments show the increase in AKT(S473) and ERK1/2(T202/Y204) phosphorylation in response to treatment with 200 ng/ml NRG-1β in MCF7 and MCF7-HER2 cells. Levels of phosphorylated AKT and ERK1/2 are comparable between cell lines both in untreated cells and after NRG-1β treatment (representative from n = 4 experimental repeats. C) MCF7-HER2 cells were treated with PI3K inhibitor LY294002 (10 μM) or dual specificity MEK kinase inhibitor PD98059 (50 μM) for 8 h before whole lysate collection. Blotting for phosphorylated ERK1/2 (T202/Y204) and AKT (S473) demonstrate the specific inhibition of these pathways by their respective inhibitors. D) OD measurements of HIF-2α protein levels in LY294002 and PD98059 treated lysates compared to equivalent vehicle (DMSO) controls in western blotting experiments shown in C. HIF-2α levels were significantly reduced after PI3K inhibition (ratio pair t-test, P < 0.05).Whilst inhibition of ERK phosphorylation led to a reduction in HIF-2α levels in all experimental repeats, this did not achieve significance (representative of n = 4 experimental repeats). (PDF 157 kb) [file 13058_2019_1097_MOESM3_ESM.pdf]

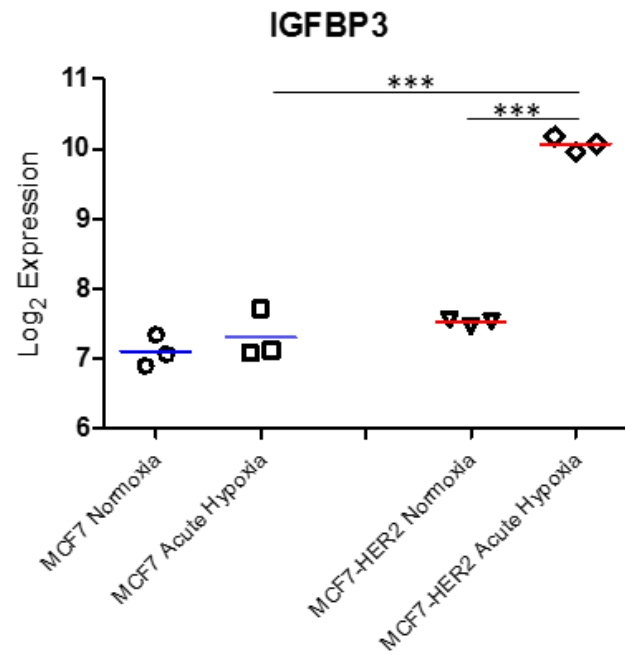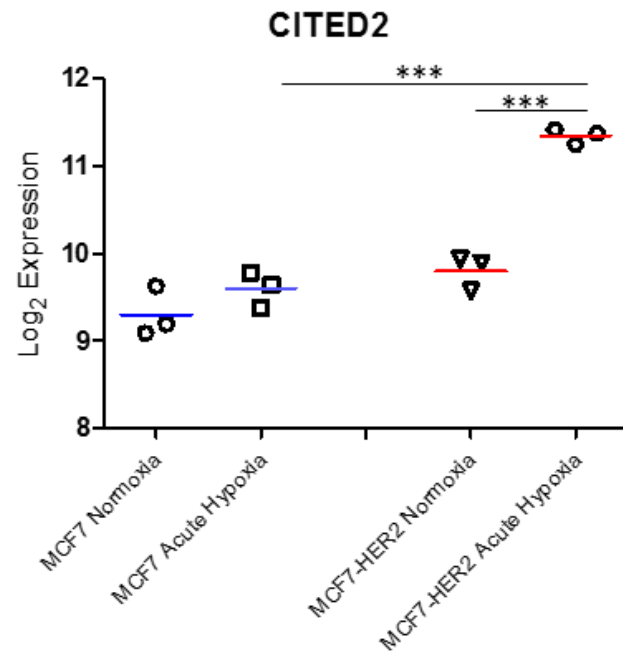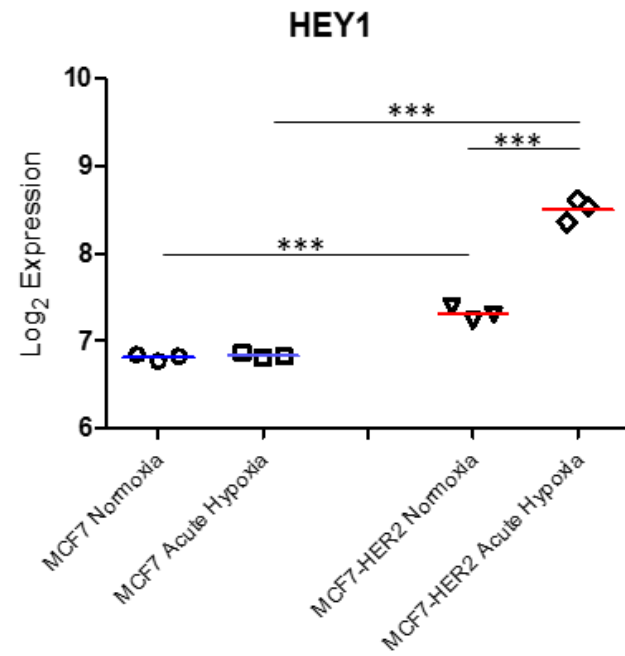

Supplement: Supplementary file 4 — Figure S4. HER2-driven hypoxic expression of HIF-2α-specific target genes. The expression levels of IGFBP3, CITED2 and HEY1 in MCF7 and MCF7-HER2 in normoxia and after exposure to acute (24 h) hypoxia in our Illumina Beadchip microarray data. Individual expression values for n = 3 repeats is shown with mean values represented by blue (MCF7) or red (MCF7-HER2) lines. These target genes have been previously demonstrated by siRNA inhibition of HIF-1α and HIF-2α to be specifically upregulated by HIF-2α in MCF7 cells [28]. The upregulation of gene expression in response to acute hypoxia is greatly facilitated by the overexpression of HER2. (ANOVA with Tukey’s multiple comparisons, *** = P < 0.001). (PDF 13 kb) [file 13058_2019_1097_MOESM4_ESM.pdf]

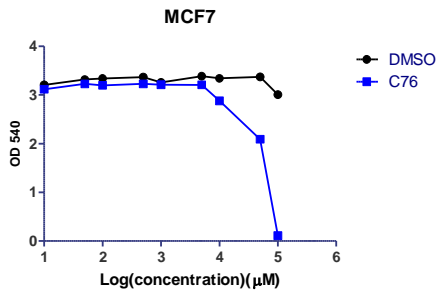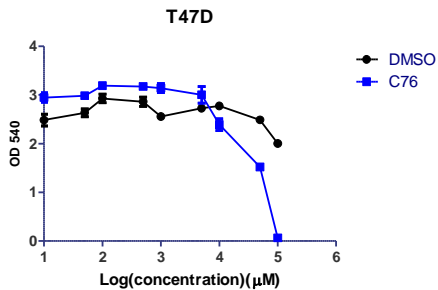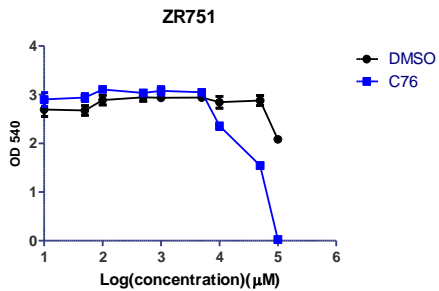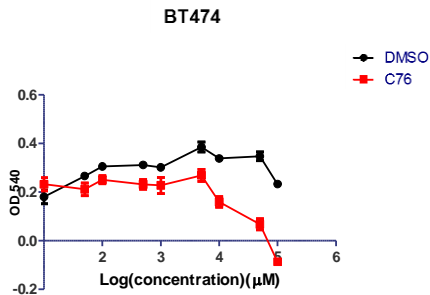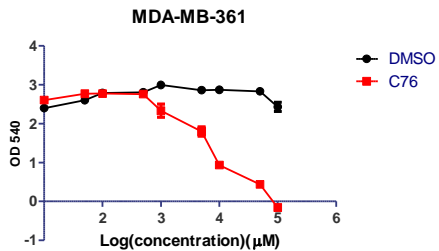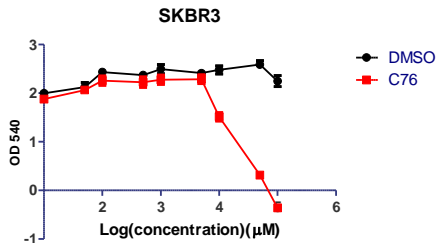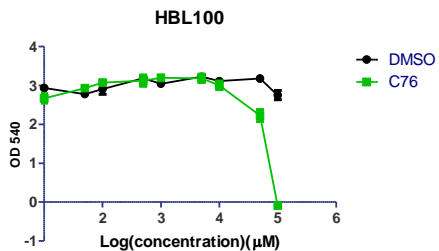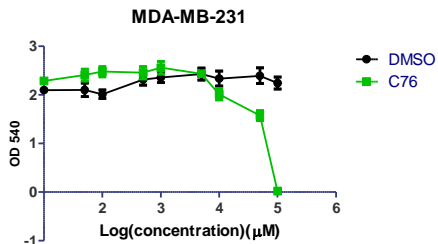

Supplement: Supplementary file 5 — Figure S5. Cell growth assays show increased sensitivity of HER2-positive cell lines to HIF-2α inhibition. Sulforhodamine B growth assays for cell lines treated with a range of concentrations of HIF-2 translation inhibitor C76 or vehicle control (DMSO). The data shown is the non-normalised version of the growth curves shown in Fig. 7D. Briefly, cells were treated with 10 nM–100 μM of C76 or equivalent volumes of DMSO for 5 days, at which point cellular density was assessed by SRB assay. Error bars represent the SEM from n = 6 repeats. (PDF 51 kb) [file 13058_2019_1097_MOESM5_ESM.pdf]

**A****All Patients**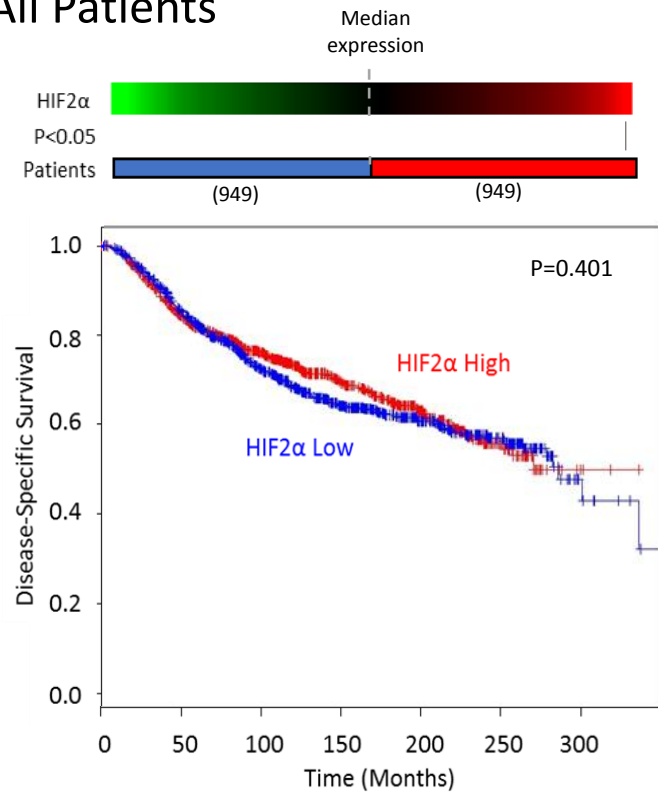**B****HER2 +**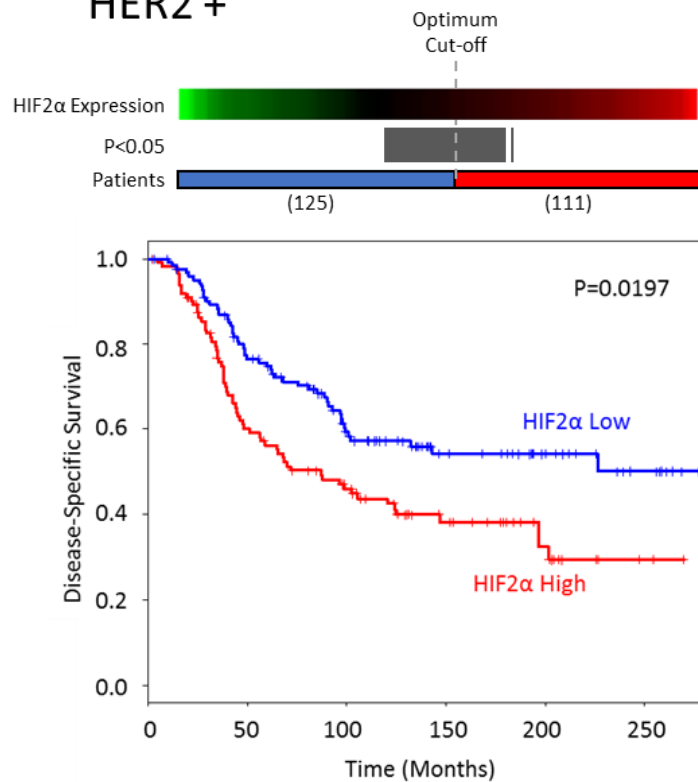**C****HER2 -**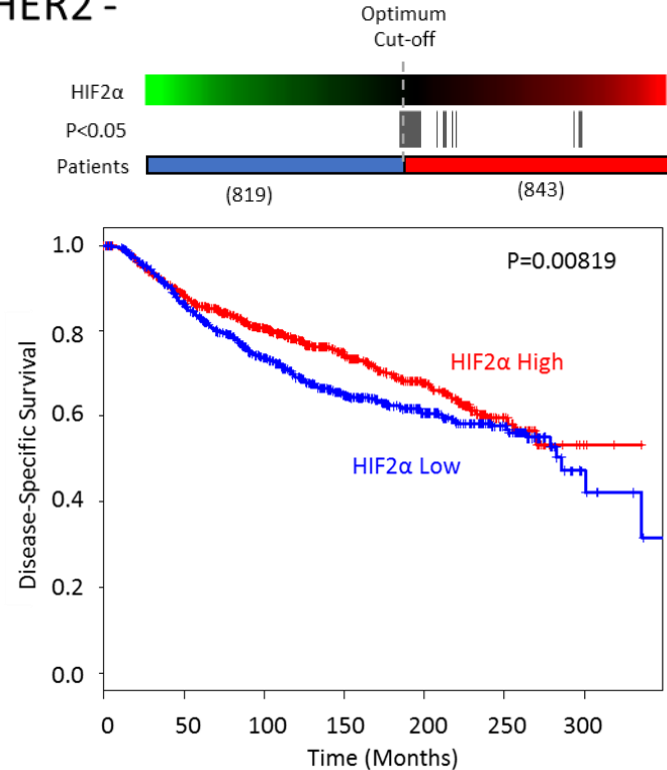

Supplement: Supplementary file 6 — Figure S6. HIF2a is associated with worse prognosis only in HER2-positive breast cancer. Kaplain-Meier plots showing disease-specific survival in the METABRIC dataset in all patients (A), HER2-positive patients (B) and HER2-negative patients (C). HIF-2α high and low categories were determined by the optimum cut-off point, whilst the complete data set used median HIF-2α expression, as no suitable significant cut-offs were present. All significant cut-off points are illustrated by grey bars above the plots, with the number of patients in each category shown as blue and red bars. HER2 positivity was determined by IHC or FISH in associated clinical metadata. HIF-2α was only significantly associated with worse prognosis in HER2-positive patients. (PDF 290 kb) [file 13058_2019_1097_MOESM6_ESM.pdf]
